# A neomorphic cancer cell-specific role of MAGE-A4 in trans-lesion synthesis

**DOI:** 10.1038/ncomms12105

**Published:** 2016-07-05

**Authors:** Yanzhe Gao, Elizabeth Mutter-Rottmayer, Alicia M. Greenwalt, Dennis Goldfarb, Feng Yan, Yang Yang, Raquel C. Martinez-Chacin, Kenneth H. Pearce, Satoshi Tateishi, Michael B. Major, Cyrus Vaziri

**Affiliations:** 1 Department of Pathology and Laboratory Medicine, University of North Carolina at Chapel Hill, 101 Manning Drive, 614 Brinkhous-Bullitt Building, Chapel Hill, North Carolina 27599, USA; 2 Curriculum in Toxicology, University of North Carolina at Chapel Hill, Chapel Hill, North Carolina 27599, USA; 3 Curriculum in Genetics and Molecular Biology, University of North Carolina, Chapel Hill, North Carolina 27599, USA; 4 Department of Computer Science, University of North Carolina at Chapel Hill, Chapel Hill, North Carolina 27599, USA; 5 Department of Cell Biology and Physiology, Lineberger Comprehensive Cancer Center, University of North Carolina at Chapel Hill, Chapel Hill, North Carolina 27599, USA; 6 Department of Pharmacology, University of North Carolina at Chapel Hill, Chapel Hill, North Carolina 27599, USA; 7 Center For Integrative Chemical Biology and Drug Discovery, Eshelman School of Pharmacy, University of North Carolina at Chapel Hill, Chapel Hill, North Carolina 27599, USA; 8 Division of Cell Maintenance, Institute of Molecular Embryology and Genetics (IMEG), Kumamoto University, Honjo 2-2-1, Kumamoto 860-0811, Japan

## Abstract

Trans-lesion synthesis (TLS) is an important DNA-damage tolerance mechanism that permits ongoing DNA synthesis in cells harbouring damaged genomes. The E3 ubiquitin ligase RAD18 activates TLS by promoting recruitment of Y-family DNA polymerases to sites of DNA-damage-induced replication fork stalling. Here we identify the cancer/testes antigen melanoma antigen-A4 (MAGE-A4) as a tumour cell-specific RAD18-binding partner and an activator of TLS. MAGE-A4 depletion from MAGE-A4-expressing cancer cells destabilizes RAD18. Conversely, ectopic expression of MAGE-A4 (in cell lines lacking endogenous MAGE-A4) promotes RAD18 stability. DNA-damage-induced mono-ubiquitination of the RAD18 substrate PCNA is attenuated by MAGE-A4 silencing. MAGE-A4-depleted cells fail to resume DNA synthesis normally following ultraviolet irradiation and accumulate γH2AX, thereby recapitulating major hallmarks of TLS deficiency. Taken together, these results demonstrate a mechanism by which reprogramming of ubiquitin signalling in cancer cells can influence DNA damage tolerance and probably contribute to an altered genomic landscape.

## Abstract (web summary)

RAD18 is an important protein in trans-lesion synthesis, an error-prone damage-tolerant mode of DNA replication. Here the authors show that MAGE-A4 stabilizes RAD18 and allows cancer cells to maintain on-going DNA synthesis in the face of genotoxic injury.

## Graphical abstract

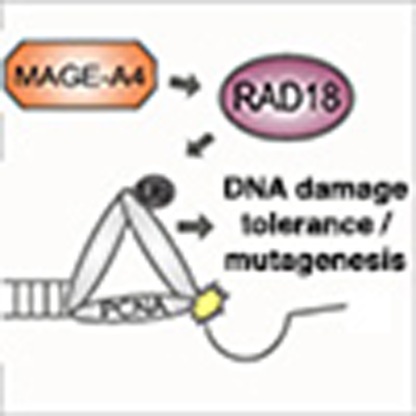

Eukaryotic cells are exposed to many intrinsic and exogenous sources of DNA damage. The S-phase of the cell cycle is particularly vulnerable to genotoxins, because error-prone replication of damaged DNA can lead to mutagenesis, a ‘hallmark and enabling characteristic' of cancer^1^. To mitigate the genome-destabilizing consequences of DNA damage in S-phase, DNA replication forks that encounter lesions trigger a network of signal transduction pathways collectively termed the DNA damage response (DDR). The different effector arms of the DDR cooperate to facilitate S-phase recovery and resumption of normal cell cycle progression following genotoxic insult^2^. Failure to integrate DNA replication with DNA repair and cell cycle progression leads to reduced viability, compromised genome stability and a predisposition to cancer.

Trans-lesion synthesis (TLS) is one of the main effector pathways of the DDR and is important for normal recovery from DNA replication fork stalling^3^. The conventional DNA polymerases that duplicate most of the genome every cell cycle cannot replicate DNA templates harbouring bulky lesions. Therefore, following acquisition of DNA damage, a ‘polymerase switch' replaces replicative DNA polymerases at stalled replication forks with specialized TLS DNA polymerases that can accommodate bulky lesions.

The Y-family TLS polymerases include DNA polymerase eta (Polη), DNA polymerase kappa (Polκ), DNA polymerase iota (Polι) and REV1 (refs 3, 4). Collectively, Y-family TLS polymerases enable cells to maintain DNA synthesis using damaged genomes. In TLS-deficient cells, checkpoint kinase signalling persists, leading to a protracted S-phase arrest and accumulation of DNA double-stranded breaks (DSBs)^5^,^6^,^7^.

TLS can be error-free or error-prone depending on the nature of the DNA damage and the particular TLS polymerase(s) selected for lesion bypass^3^,^4^. Polη is the default TLS polymerase recruited to stalled replication forks and performs error-free replication of DNA templates containing its cognate lesions (including ultraviolet-induced cyclo-butane pyrimidine dimers), thereby suppressing mutagenesis. However, when Polη is absent, error-prone compensatory lesion bypass by other Y-family DNA polymerases leads to mutations^8^, a mechanism that explains the ultraviolet sensitivity and skin cancer propensity of Polη-deficient xeroderma pigmentosum-Variant patients^9^. TLS must be regulated strictly and used sparingly to ensure genomic stability.

Mono-ubiquitination of the DNA polymerase processivity factor proliferating cell nuclear antigen (PCNA) is important for TLS activation and lesion bypass^10^,^11^. In response to DNA damage, the E3 ubiquitin ligase RAD18 is recruited to stalled replication forks where it mono-ubiquitinates PCNA at the conserved residue K164 (refs 12, 13). K164 mono-ubiquitination promotes interactions between PCNA and Y-family TLS polymerases (which possess ubiquitin-binding zinc fingers and ubiquitin-binding motifs) at stalled replication forks^14^.

RAD18 overexpression can increase PCNA mono-ubiquitination and promote recruitment of TLS polymerases to replication forks, even in the absence of DNA damage^5^. Conversely, in *RAD18*-deficient cells, Y-family TLS polymerases are not recruited efficiently to sites of DNA replication stalling^5^,^15^,^16^ and overall lesion bypass is reduced^17^. Moreover, RAD18 deficiency recapitulates the defective S-phase recovery phenotypes of Polη and Polκ-deficient cells after genotoxin exposure^5^, supporting a major role for RAD18 in TLS.

Although RAD18 is important for TLS polymerase recruitment to stalled replication forks, the basis for lesion-specific selection of the correct TLS polymerase is not yet fully understood. All TLS polymerases preferentially associate with mono-ubiquitinated PCNA relative to unmodified species. Clearly, relative expression levels and activities of RAD18 and the Y-family DNA polymerases are likely to have an impact on the overall TLS capacity and accuracy, determining mutagenic outcomes. Recent sequencing efforts have demonstrated that cancer cell genomes contain tens to hundreds of thousands of nucleotide substitutions and other mutations^18^. Mutation rates of untransformed cells are insufficient to explain the large numbers of mutations found in cancer cells. Therefore, cancer may be associated with a ‘mutator phenotype' that generates large numbers of driver and passenger mutations during tumour progression^19^,^20^. Owing to its pivotal role in error-prone DNA synthesis, RAD18-mediated TLS has the potential to contribute to the mutational burden of cancer genomes. Neoplastic cells experience various oncogene-induced forms of DNA damage and replication stress (including oxidative DNA damage from reactive oxygen species (ROS) and re-replication) throughout tumour progression. The ATR/CHK1 branch of the DDR may help pre-neoplastic cells endure oncogenic stress, thereby promoting tumorigenesis^21^. Similarly, the RAD18–TLS pathway is in essence a DNA-damage tolerance mechanism that could help maintain viability in the face of oncogene-induced replication stresses^22^. Therefore, RAD18–TLS has the potential to have an impact on tumorigenesis by promoting error-prone DNA synthesis and by conferring oncogenic stress tolerance. However, whether dysregulation of the TLS pathway has an impact on genome maintenance mechanisms and phenotypes of cancer cells is unknown. Most of our understanding of the mammalian RAD18–TLS signalling pathway stems from studies performed in cultured cancer cell lines. Remarkably, however, it is unknown whether RAD18 and TLS are differentially regulated in cancer cell lines and untransformed cells.

In this report we identify a cancer cell-specific protein, the cancer/testes antigen (CTA) melanoma antigen-A4 (MAGE-A4), as a novel binding partner and stabilizing factor for RAD18. CTA proteins are ordinarily germ line restricted, yet can be aberrantly expressed at high levels in many cancers^23^. The MAGE represent a subclass of CTA^24^, some of which were recently shown to associate with and activate specific RING E3 ubiquitin ligases^25^, thereby providing a new mechanism by which ubiquitin signalling is deregulated in cancer cells. Here we show that MAGE-A4 contributes to TLS pathway activation, DNA-damage tolerance and genome maintenance in cancer cells. These results suggest a mechanism by which cancer genomes are impacted via reprograming of ubiquitin signalling.

## Results

### MAGE-A4 is a component of the RAD18–RAD6 complex

**Figure 1:**
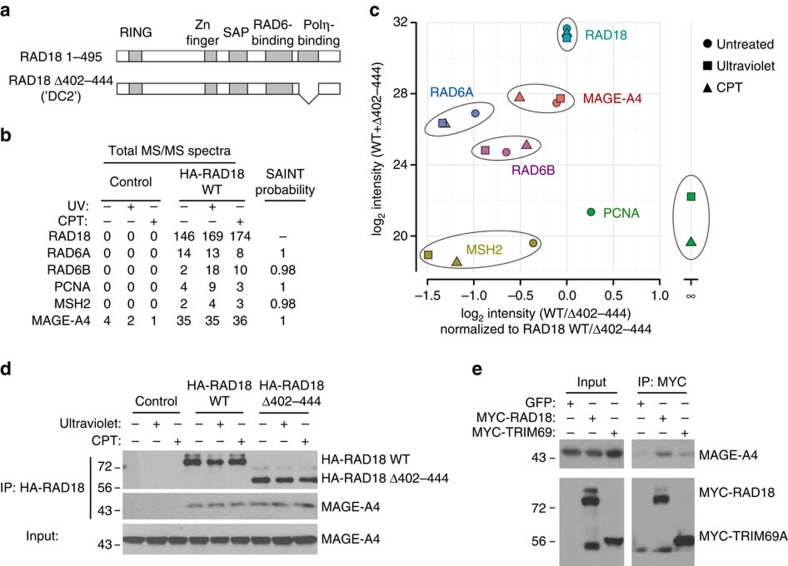
MAGE-A4 is a novel component of the RAD18 complex in cancer cells. (**a**) Domain organization of full-length RAD18 and RAD18 Δ402–444 (which harbours an internal deletion removing the Polη-binding domain). (**b**) Spectral counts and estimated probability of true interaction by SAINT analysis for selected proteins identified in HA–RAD18-WT and Control (HA) APMS experiments. (**c**) Total protein signal intensity versus relative abundance between HA–RAD18-WT and HA–RAD18 Δ402–444 APMS. Signal intensity was normalized to the corresponding experiment's bait intensity (*x* axis). (**d**) H1299 cells were infected with adenoviruses encoding WT HA–RAD18, HA–RAD18 Δ402–444 or with an ‘empty' control adenovirus. Infected cells were treated with CPT (2 μM) or UVC (20 J m^−2^). Two hours (h) later, cell extracts were prepared and immunoprecipitated with anti-HA antibody-conjugated magnetic beads. The resulting immune complexes and input fractions were analysed by immunoblotting with anti-HA and anti-MAGE-A4 antibodies. (**e**) Expression vectors encoding MYC–RAD18, MYC–TRIM69 or green fluorescent protein (GFP) (for control plasmid) were transiently transfected into H1299 cells. Extracts from the resulting cells were immunoprecipitated with an anti-MYC antibody and the resulting immune complexes (or input fractions) were analysed by immunoblotting with antibodies against MAGE-A4 and MYC.

To identify new regulators of the TLS pathway we defined the RAD18 protein interaction network in H1299 adenocarcinoma cells using label-free affinity purification and shotgun mass spectrometry (APMS). As a control we also investigated the protein interaction network of a TLS-compromised RAD18 Δ402-444 mutant, which lacks a domain involved in mediating binding to Polη^26^,^27^ and other partners^28^ (Fig. 1a). RAD18 interaction networks were defined for HA–RAD18 wild type (WT) and HA–RAD18 Δ402-444 complexes isolated from undamaged cells and from genotoxin (ultraviolet or camptothecin (CPT))-treated cultures.

Co-complexed proteins were separated from background contaminants and false positives using the SAINT (significance analysis of interactome) algorithm (Supplementary Data 1). Top-scoring proteins included well-known RAD18 interactors such as RAD6A and RAD6B (E2 ubiquitin-conjugating enzymes), PCNA (a RAD18 substrate) and MSH2, a reported regulator of RAD18 (ref. 29; Fig. 1b). As expected from previous work^30^, PCNA binding to RAD18 Δ402-444 was decreased or undetectable (Fig. 1c and Supplementary Note 1). The complete list of proteins detected in RAD18 complexes is available in Supplementary Data 1. One of the highest confidence and abundant novel RAD18 interactors we identified was the CTA MAGE-A4 (Fig. 1c).

The presence of MAGE-A4 in the RAD18 complexes was unaffected by ultraviolet or CPT, genotoxins that activate the distinct TLS and DSB repair effector pathways of RAD18, respectively. Comparison of relative MAGE-A4 abundance between RAD18 WT and RAD18 Δ402-444 APMS revealed that MAGE-A4 association does not depend on the Polη-binding domain of RAD18.

As the association of RAD18 with MAGE-A4 provided a potentially important new relationship between DNA-damage tolerance and cancer, we validated and further characterized the RAD18–MAGE-A4 interaction. First, we confirmed the RAD18–MAGE-A4 interaction in H1299 cells by performing independent co-immunoprecipitation (co-IP) and immunoblotting experiments (Fig. 1d).

A genome-wide screen previously detected MAGE-A4 as a binding partner of the E3 ubiquitin ligase TRIM69 (ref. 31), a mitotic regulator^32^. For the purpose of comparison with a known MAGE-A4 partner, we expressed MYC epitope-tagged RAD18 and TRIM69 at similar levels in H1299 cells and examined levels of MAGE-A4 associated with each E3 ligase by co-IP. In a side-by-side comparison, RAD18 immune complexes contained more MAGE-A4 than was present in TRIM69 immunoprecipitates (Fig. 1e). Other E3 ubiquitin ligases we tested (HLTF1, SHPRH, RNF8 and RNF168) failed to co-IP with MAGE-A4 (not shown). We conclude that MAGE-A4 is a specific and constitutive component of the RAD18 complex in H1299 lung carcinoma cells.

### MAGE-A4 associates with the RAD6-binding domain of RAD18

**Figure 2:**
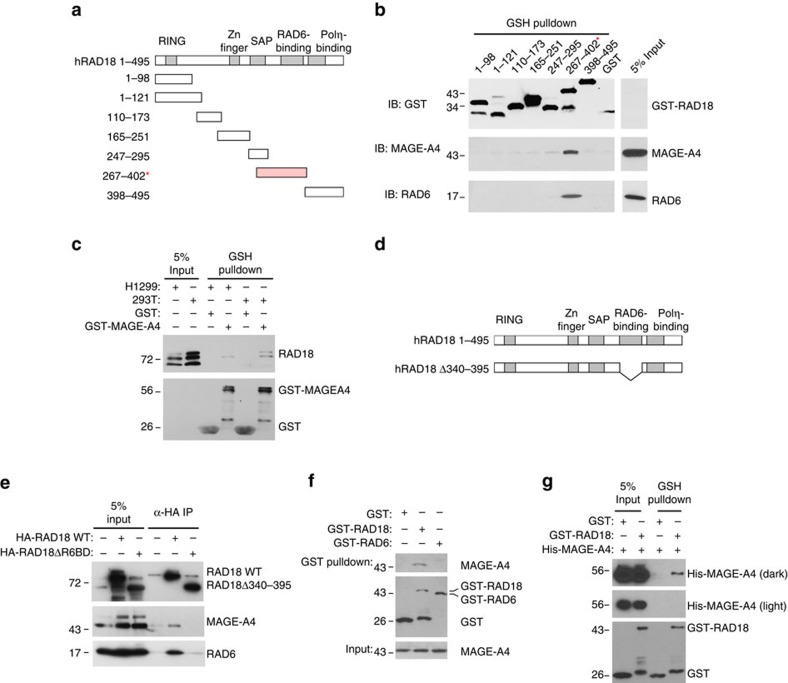
MAGE-A4 associates with the RAD6-binding domain of RAD18. (**a**) The indicated RAD18 fragments were expressed as GST fusions in *E. coli*. The RAD6-binding domain spanning residues 267–402 is highlighted in red. (**b**) GST–RAD18 fragments were incubated with H1299 cell extracts. After ‘pull-down' with GSH-sepharose beads, the recovered GST–RAD18 fusions and 5% of ‘input' H1299 cell lysate were analysed by immunoblotting with antibodies against GST, MAGE-A4 and RAD6. (**c**) GST–MAGE-A4 or GST was incubated with extracts from H1299 or 293T cells. After pulldown with GSH-sepharose beads, the recovered GST proteins (and 5% of input cell extract) were analysed by immunoblotting with antibodies against GST and RAD18. (**d**) Domain organization of full-length RAD18 and the RAD18 Δ340–395 (ΔR6BD) mutant harbouring an internal deletion that removes the RAD6-binding domain. (**e**) H1299 cells were transiently transfected with expression plasmids encoding HA–RAD18 and HA–RAD18 Δ340–395 (ΔR6BD) or with an empty vector control. Lysates from the resulting cells were immunoprecipitated with anti-HA antibodies. Anti-HA immune complexes and inputs (20 μg) were analysed by immunoblotting with antibodies against RAD18, MAGE-A4 and RAD6. (**f**) Recombinant GST, GST–RAD18 267–402 or GST–RAD6 were incubated with H1299 cell extracts then pulled down with GSH-sepharose beads. The recovered GST proteins were analysed by immunoblotting with antibodies against MAGE-A4 and GST. (**g**) Recombinant GST and GST–RAD18 267–402 were incubated with full-length recombinant Hexa-histidine-tagged MAGE-A4 (His-MAGE-A4). GST proteins were recovered using GSH-sepharose beads. Recovered GST proteins (and 5% of input) were analysed by immunoblotting with antibodies against GST and MAGE-A4.

E3 ubiquitin ligases share many common sequence motifs. However, for the known MAGE-interacting RING-domain E3 ligases, no single consensus sequence or domain of the E3 is sufficient to mediate MAGE binding^24^,^25^. Therefore, we performed experiments to map the MAGE-A4-interacting domain of RAD18. We expressed the different functional domains of the RAD18 protein as individual in-frame fusions with glutathione *S*-transferase (GST) (Fig. 2a), then performed ‘pull-down' assays to identify the MAGE-A4-binding domain(s) of RAD18. As shown in Fig. 2b, the GST–RAD18 267-402 fragment, specifically recovered MAGE-A4 from H1299 cell lysates. In reciprocal ‘pull-down' experiments, GST–MAGE-A4 also recovered RAD18 from H1299 and 293T cell lysates (Fig. 2c).

Interestingly, GST–RAD18 (267–402) contains the RAD6-binding domain (amino acids 340–395) previously defined by Watanabe *et al*.^26^. Similar to MAGE-A4, RAD6 was only recovered from cell lysates with GST–RAD18 (267–402) (Fig. 2b). To determine whether the RAD6-binding domain is also involved in RAD18–MAGE-A4 complex formation in cells, we determined the effect of internal deletion of amino acids 340–395 on the RAD18–MAGE-A4 association. Using transient transfection, HA–RAD18 (WT) and HA–RAD18 Δ340–395 (Fig. 2d) were expressed at similar levels in H1299 cells (Fig. 2e). However, in co-IP and immunoblotting experiments, MAGE-A4 and RAD6 only associated with WT RAD18 (Fig. 2e). We conclude that the RAD6-binding domain is necessary for RAD18–MAGE-A4 interactions *in vitro* and in cells.

We considered the possibility that the association of MAGE-A4 with RAD18 might be indirect and mediated via RAD6. However, in pull-down experiments recombinant GST–RAD6 did not recover MAGE-A4 from H1299 cell lysates (Fig. 2f). To more carefully evaluate a role for RAD6 (or other factors) in mediating the RAD18–MAGE-A4 interaction, we performed binding studies using purified MAGE-A4 and GST–RAD18 (267–402). As shown in Fig. 2g, we detected specific association of RAD18 (267–402) with MAGE-A4 in the absence of RAD6. Using ALPHAscreen-based protein proximity assays^33^, we independently validated the association of isolated MAGE-A4 (and of RAD6) with RAD18 (267–402) (Supplementary Fig. 1). Interestingly, recombinant unlabelled RAD6 competed with epitope-tagged MAGE-A4 for RAD18 binding both *in vitro* and in cells (Supplementary Fig. 1a–d). However, gel filtration chromatography experiments show that most of the cellular RAD6 is free and monomeric (Supplementary Fig 1e–h). Moreover, from quantitative immunoblotting, RAD6 levels in H1299 cells exceed MAGE-A4 by 28-fold and exceed RAD18 levels by 114-fold (Supplementary Fig. 2). Therefore, MAGE-A4 is not sufficiently abundant in H1299 cells to outcompete RAD6 for RAD18 association. We conclude that MAGE-A4 is a specific binding partner of RAD18 and associates with the RAD6-binding domain (as also reported for p95/NBS1)^34^.

### MAGE-A4 promotes RAD18 stability

**Figure 3:**
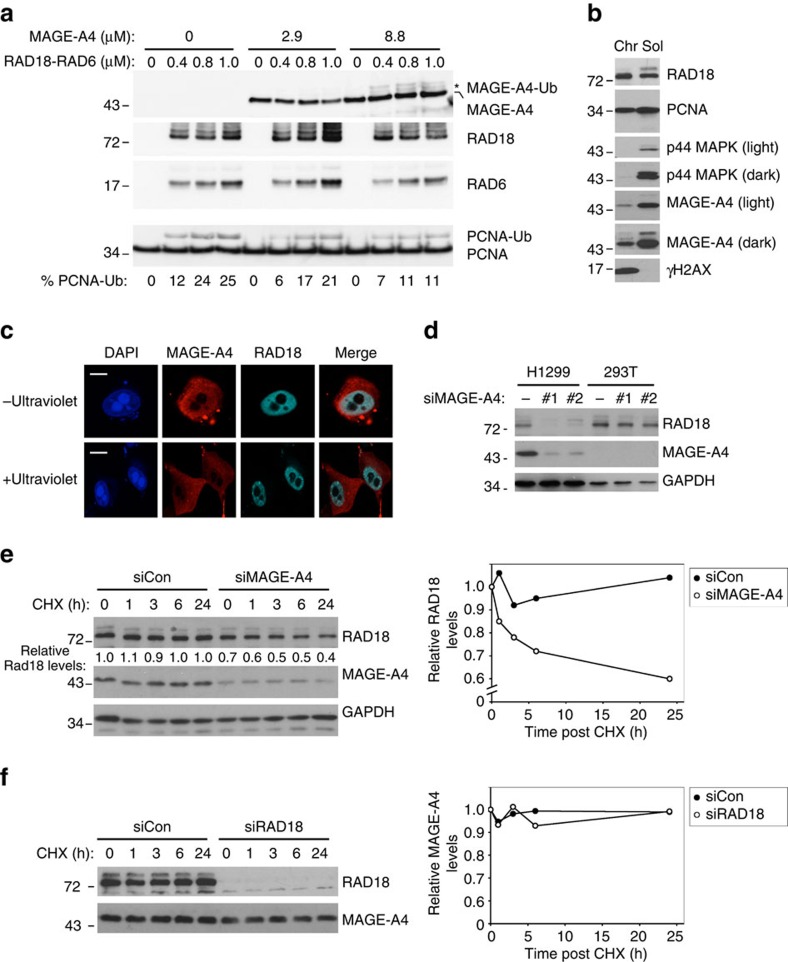
MAGE-A4 promotes RAD18 stability. (**a**) Recombinant RAD18–RAD6 complex (0, 0.27, 0.54 and 0.82 μM) was incubated with E1, ubiquitin and purified PCNA. Reaction products were analysed by immunoblotting with antibodies against the indicated proteins. (**b**) Soluble and chromatin fractions from H1299 cells were analysed by SDS–PAGE (20 μg per lane) and immunoblotting with antibodies against the indicated proteins. (**c**) H1299 cells were transiently transfected with an expression plasmid encoding CFP-RAD18 (or empty vector for control), ultraviolet irradiated (20 J m^−2^) and processed for immunofluorescence microscopy after 6 h. Scale bar, 10 μm. (**d**) H1299 and 293T cells were transfected with two independent siRNAs targeting MAGE-A4 or with control non-targeting siRNA oligonucleotides. After 72 h, extracts from the siRNA-transfected cells were analysed by immunoblotting with antibodies against the indicated proteins. (**e**,**f**) H1299 cells were transfected with siRNA oligonucleotides against MAGE-A4, RAD18 or control non-targeting siRNA as indicated. Forty eight hours later, cells were treated with cycloheximide (CHX, 100 μg ml^−1^) and collected at different time points for immunoblot analysis.

Reportedly, several MAGE family members directly activate their partner E3 ligases to promote substrate ubiquitination^25^. Therefore, we performed *in vitro* ubiquitin ligase assays using recombinant proteins, to determine the effect of MAGE-A4 on RAD18-directed PCNA mono-ubiquitination. As shown in Fig. 3a, recombinant MAGE-A4 did not stimulate RAD18-dependent PCNA mono-ubiquitination under experimental conditions where other MAGE proteins stimulate catalytic activities of their cognate E3 ligases^25^. Interestingly, MAGE-A4 was ubiquitinated by RAD18 (Fig. 3a). High molar ratios of MAGE-A4:RAD18 led to decreased PCNA mono-ubiquitination *in vitro* (Fig. 3a). The apparent mild inhibition of PCNA mono-ubiquitination by MAGE-A4 *in vitro* results from substrate competition when MAGE-A4 is in vast excess of PCNA (Fig. 3a, lanes 9–12).

The major substrate and distal effector of RAD18-mediated ubiquitination in DNA damage tolerance is the sliding clamp PCNA, which is present on replicating chromatin in the nucleus.

Although PCNA and RAD18 were present in both chromatin and soluble fractions, MAGE-A4 was primarily soluble (Fig. 3b). Moreover, in ultraviolet-irradiated H1299 cells, RAD18 but not MAGE-A4 redistributed to nuclear foci representing sites of DNA replication stalling (Fig. 3c and Supplementary Fig. 3). Taken together, the results of Fig. 3a–c suggest that MAGE-A4 may not function as an allosteric activator of RAD18 or respond directly to replication fork stalling. Accordingly, we investigated alternative roles for MAGE-A4 in RAD18 regulation.

Proteins often stabilize their binding partners. Therefore, we determined the effect of MAGE-A4 depletion on RAD18 levels. As shown in Fig. 3d, we attained ∼90% depletion of MAGE-A4 in H1299 cells using two independent transiently transfected small interfering RNAs (siRNAs). Interestingly, both MAGE-A4-directed siRNAs led to substantial (92% and 73% decreases in RAD18 expression in H1299 cells). Neither MAGE-A4-directed siRNA affected RAD18 levels in 293T cells, which lack detectable MAGE-A4 expression (Fig. 3d). Using cycloheximide treatment to block new protein synthesis, we measured RAD18 decay rates in control and MAGE-A4-depleted cultures. In control (MAGE-A4 replete) H1299 cells, RAD18 was stable for at least 24 h (the duration of this experiment, see Fig. 3e). In MAGE-A4-depleted cells, RAD18 expression was reduced and its half-life decreased when compared with MAGE-A4-replete cells (Fig. 3e). RAD18 depletion did not affect the half-life of MAGE-A4 (Fig. 3f). However, we note that MAGE-A4 levels exceed those of RAD18 by ∼3-fold in H1299 cells (Supplementary Fig. 2). Moreover, most of the cellular MAGE-A4 is not nuclear (Fig. 3c) or in the same complex as RAD18 (Supplementary Fig. 1f–h), explaining why RAD18 does not influence the overall MAGE-A4 pool.

**Figure 4:**
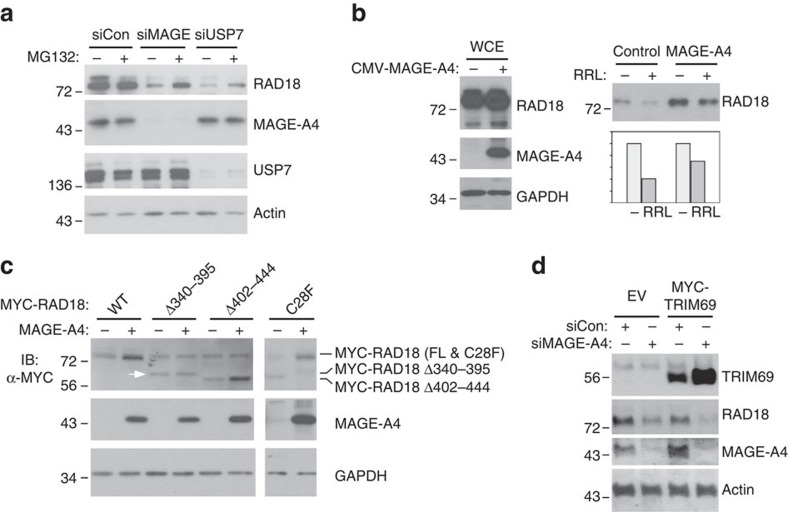
MAGE-A4 protects RAD18 from ubiquitin-mediated proteolysis. (**a**) Replicate plates of H1299 cells were transfected with siRNA against MAGE-A4, USP7 or with non-targeting control siRNA. After 48 h, one plate of each replicate was treated with 10 μM MG132 for 16 h. Extracts from control and MG132-treated cells were analysed by immunoblotting with antibodies against the indicated proteins. (**b**) 293T cells were co-transfected with an HA–RAD18 expression vector in combination with a CMV-MAGE-A4 plasmid or an empty vector for control. After 48 h, RAD18 complexes were immunoprecipitated with anti-HA antibodies. The resulting immune complexes were incubated in a rabbit reticulocyte lysate (RRL) to reconstitute ubiquitin-coupled proteolysis *in vitro*. Relative levels of RAD18 and MAGE-A4 were determined by immunoblotting and quantified using densitometry. (**c**) H1299 cells were transiently co-transfected with WT or mutant HA–RAD18 expression plasmids in combination with a MAGE-A4 expression vector (or empty vector control). Forty eight hours later, cells were harvested for immunoblot analysis of RAD18 and MAGE-A4. The white arrowhead indicates the RAD18Δ340–395 mutant protein band that is insensitive to MAGE-A4. (**d**) Replicate cultures of H1299 cells were transfected with an expression vector encoding MYC–TRIM69 or with an empty vector plasmid for control. Sixteen hours later, the cells were transfected with siRNA against MAGE-A4 or with a scrambled control siRNA and incubated for an additional 48 h before immunoblot analysis.

Figure 3d–f suggested that MAGE-A4 stabilizes RAD18. In previous work, proteasomal degradation of RAD18 (in USP7-depleted cells) was partially prevented by treatment with the proteasome inhibitor MG132 (ref. 35). Therefore, we determined the effect of MG132 treatments on RAD18 stability in control (MAGE-A4 replete), MAGE-A4-depleted and USP7-depleted H1299 cells. RAD18 levels were unaffected by MG132 in MAGE-A4-replete H1299 cells in which RAD18 is stable and has a half-life (*t*_1/2_) exceeding 24 h (Fig. 3e). However, the reduced RAD18 stability in USP7- or MAGE-A4-depeleted H1299 cells was partially rescued by MG132 treatment (Fig. 4a). MG132-induced poly-ubiquitin laddering of RAD18 was also decreased by ectopically expressed MAGE-A4 in 293T cells, which lack endogenous MAGE-A4 (Supplementary Fig. 4). To further test the effect of MAGE-A4 on RAD18 stability, we reconstituted the ubiquitin-coupled proteolysis of RAD18 in a cell-free rabbit reticulocyte lysate and compared the degradation of immunopurified HA–RAD18 complexes from control and MAGE-A4-expressing cells. As shown in Fig. 4b, HA–RAD18 derived from MAGE-A4 co-expressing 293T cells was degraded less efficiently when compared with RAD18 from control cultures lacking endogenous MAGE-A4. Taken together Figs 3a–f and 4a,b show that MAGE-A4 protects RAD18 from ubiquitin-coupled proteolysis.

The results of Fig. 2 suggest that MAGE-A4 increases RAD18 expression via direct binding. Therefore, we compared the stabilizing effects of co-transfected MAGE-A4 on HA–RAD18 WT and the MAGE-A4-interaction-deficient HA–RAD18 Δ340–395 mutant. As shown in Fig. 4c, levels of HA–RAD18 WT were increased by co-expressed MAGE-A4. HA–RAD18 Δ402–444 (which is defective for Polη interaction but binds MAGE-A4) was also stabilized by co-expressed MAGE-A4. However, levels of HA–RAD18 Δ340–395 (indicated by the white arrowhead in Fig. 4c) were insensitive to MAGE-A4.

The MAGE-A4-interaction-deficient RAD18 mutant also lacks RAD6-binding activity. Therefore, we considered the possibility that failure of MAGE-A4 to stabilize RAD18 Δ340–395 was secondary to impaired ubiquitin ligase activity. However, a catalytically inactive RAD18 C28F mutant was stabilized by co-expressed MAGE-A4 (Fig. 4c). We conclude that MAGE-A4 stabilizes RAD18 via direct interactions with the RAD6-binding motif and independently of RAD18 E3 ligase activity.

Next we asked whether stabilization of associated E3 ligases represents a general mechanism for modulation of ubiquitin signalling by MAGE-A4. We determined the effect of MAGE-A4 expression on TRIM69 levels. As shown in Fig. 4d, MAGE-A4 expression was inversely correlated with TRIM69 levels. Therefore, the stabilizing effect of MAGE-A4 on RAD18 expression is relatively specific. Other MAGE-A4-associated E3 ligases have not been identified but eventually it will be interesting to elucidate the basis for the differential effects of MAGE-A4 on stability of its (putative) other E3 ligase partners.

### Structural basis for MAGE-induced RAD18 stability

**Figure 5:**
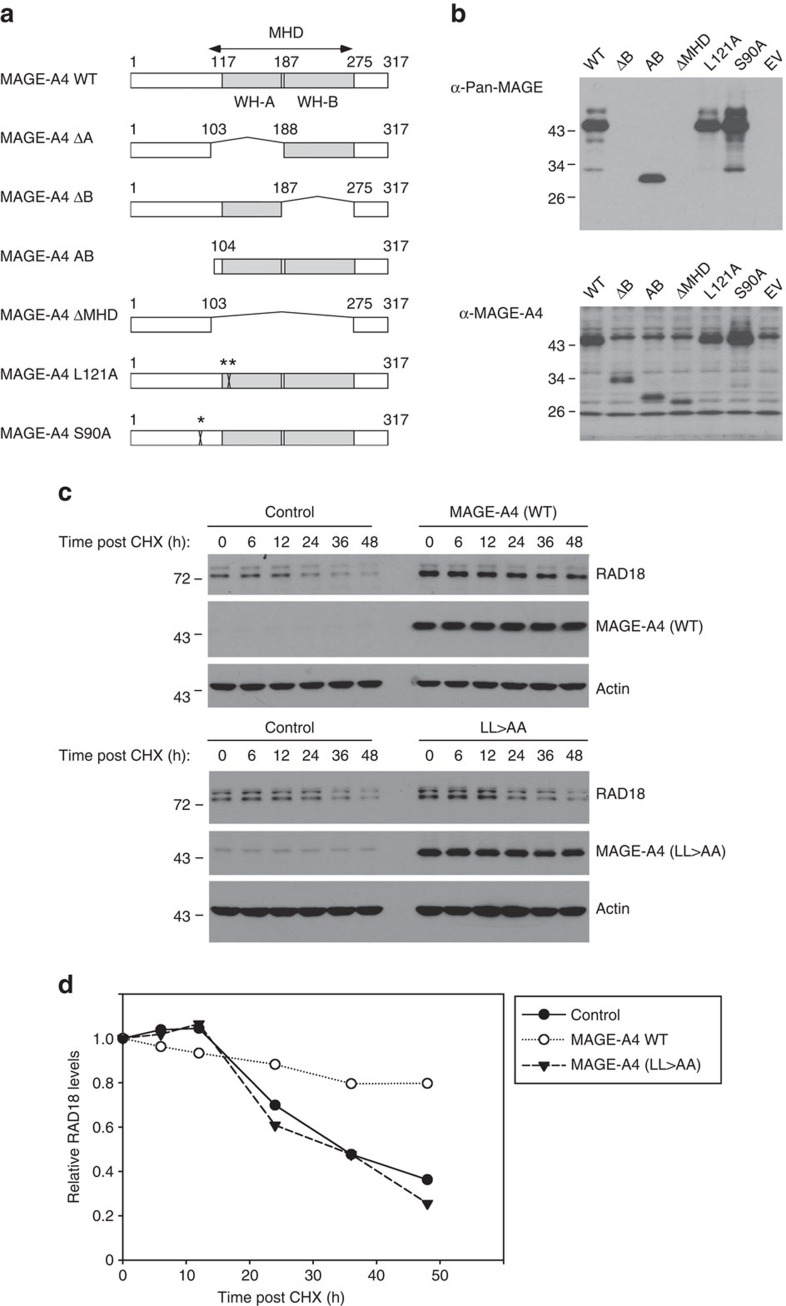
Mutational analyses to define structural requirements for MAGE-A4-induced RAD18 stabilization. (**a**) Domain structure of full-length MAGE-A4 and MAGE-A4 mutants used in this study. The MAGE-homology domain (MHD) is conserved between MAGE family members and comprises juxtaposed WH-A and WH-B regions. (**b**) 293T cells were transiently transfected with expression vectors encoding the MAGE-A4 mutants shown in **a** or with an empty vector (EV). After 48 h, extracts from the resulting cells were analysed by immunoblotting with anti-Pan-MAGE-A (which recognizes an epitope in the WH-B domain) or with anti-MAGE-A4 (which recognizes a C-terminal epitope of MAGE-A4 in residues 275–317). (**c**) Replicate plates of 293T cells were transiently transfected with expression vectors encoding WT or mutant forms of MAGE-A4. Forty-eight hours post transfection, cells were treated with cycloheximide (CHX) and then harvested at different times post CHX. Cell extracts were analysed by immunoblotting with antibodies against RAD18, MAGE-A4 and actin. (**d**) RAD18 levels in each lane of immunoblots in **c** were quantified by densitometry with ImageJ software. The graph indicates the levels of RAD18 remaining at each time point following CHX treatment in control and MAGE-A4-expressing cells.

Previous investigators have used deletion and truncation mutants to isolate separable functional domains of MAGE proteins (albeit for effectors other than RAD18)^36^,^37^. Therefore, we performed structure–function analyses to define MAGE-A4 residues and domains that are important for stabilizing RAD18. We generated MAGE-A4 deletion mutants lacking or retaining the winged-helix (WH)-A and WH-B regions of the MAGE-homology domain, as illustrated in Fig. 5a. In addition, we generated a MAGE-A4 LL>AA mutant harbouring alanine substitutions in a di-Leucine motif (L121 and L122) that is conserved between MAGE proteins and is generally necessary for their interactions with E3 ubiquitin ligase partners. We also generated a MAGE-A4 mutant with an alanine substitution at Serine 90, a phosphorylated residue present in RAD18-associated MAGE-A4 (Supplementary Data 1). In transient transfections, the MAGE-A4 mutants were expressed with different efficiencies in 293T cells. Most notably, mutants lacking the WH-A and WH-B domains expressed poorly when compared with full-length MAGE-A4 (Fig. 5b and Supplementary Fig. 5). We compared the various MAGE-A4 mutants for RAD18-stabilizing activity. As expected, WT MAGE-A4 extended the half-life of RAD18 from ∼24 to >50 h in 293T cells (Fig. 5c,d). MAGE-A4 S90A retained RAD18-stabilizing activity, indicating that MAGE-A4 S90 phosphorylation is dispensable for regulating RAD18 expression levels (Supplementary Fig. 5). MAGE-A4 LL>AA did not affect RAD18 levels, suggesting that MAGE-A4–RAD18 interactions are necessary for MAGE-A4 to stabilize RAD18.

All MAGE-A4 deletion mutants (including MAGE-A4 mutant AB, which retains a pro-apoptotic carboxy-terminal domain of MAGE-A4 previously shown to bind gankyrin^36^,^37^) failed to stabilize RAD18. We conclude that the individual WH-A or WH-B domains, or the entire MAGE-homology domain and its flanking sequences alone are insufficient to confer RAD18 stability. Instead, it is most likely to be that multiple regions of the MAGE-A4 protein act in a concerted non-separable manner to stabilize RAD18.

**Figure 6:**
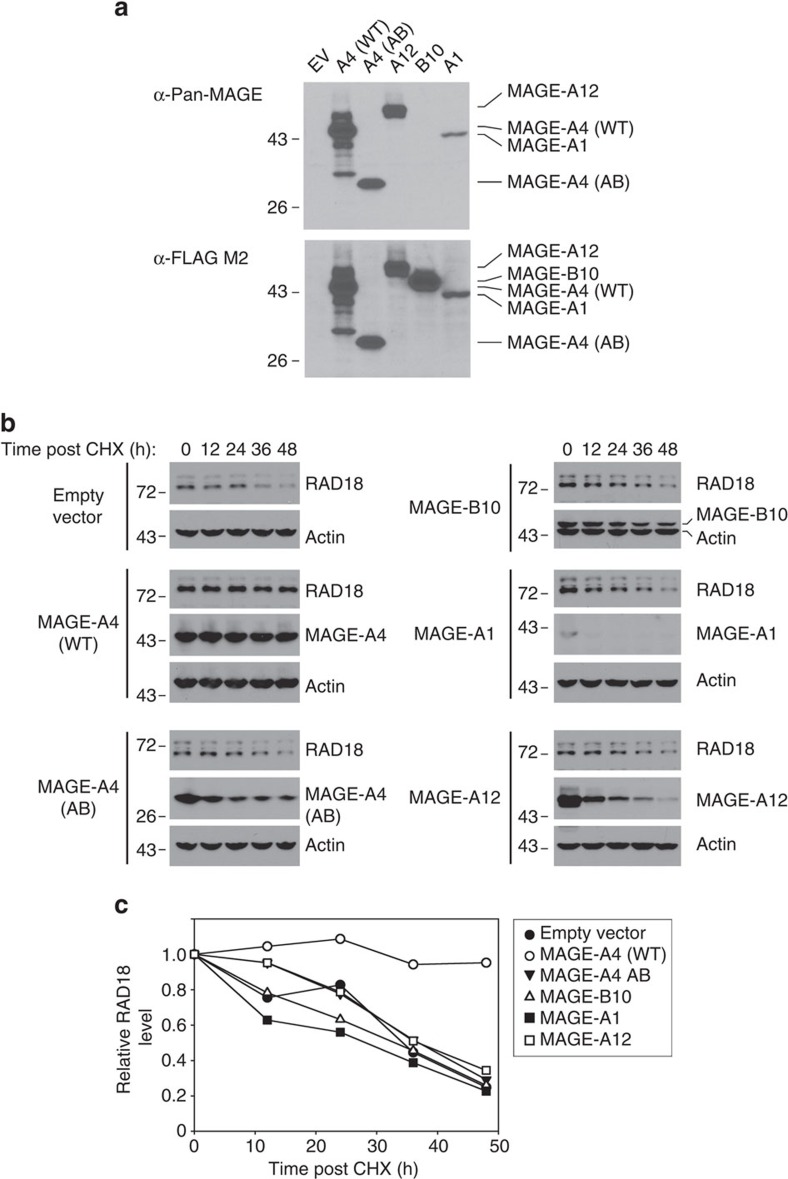
Effect of MAGE family members on RAD18 stability. (**a**) 293T cells were transfected with expression vectors encoding FLAG-tagged forms of WT MAGE-A4, MAGE-A4 AB (see Fig. 5a), MAGE-A12, MAGE-B10 and MAGE-A1. After 48 h, extracts were prepared from the transfected cells and analysed by immunoblotting with anti-Pan-MAGE and anti-FLAG antibodies. (**b**) Replicate plates of 293T cells were transiently transfected with expression vectors encoding WT MAGE-A4, MAGE-A4 AB, MAGE-A12, MAGE-B10 and MAGE-A1. Forty-eight hours post transfection, cells were treated with cycloheximide (CHX) and then harvested at different times post CHX. Cell extracts were analysed by immunoblotting with antibodies against RAD18, FLAG and actin. (**c**) RAD18 levels in each lane of immunoblots in **b** were quantified by densitometry with ImageJ software. The graph indicates the levels of RAD18 remaining at each time point following CHX treatment in control and MAGE-expressing cells.

The MAGE family members are highly conserved and may, in some cases, have overlapping functions in activating their E3 ligase partners^25^. It was of interest to determine the extent to which other MAGE family members stabilized RAD18. We were able to ectopically express MAGE-A12, MAGE-B10 and MAGE-A1 in 293T cells (Fig. 6a) and therefore these particular CTAs were tested for RAD18-stabilizing activity. Unexpectedly, despite the high conservation of primary sequences and domains between different MAGE family members, only MAGE-A4 stabilized RAD18 (Fig. 6b,c). Interestingly, these cycloheximide stability experiments also showed that MAGE-A4 has a long half-life (>48 h) when compared with MAGE-B10, MAGE-A1 and MAGE-A12. Therefore, MAGE-A4 is highly stable compared with other MAGE family members and specifically stabilizes RAD18.

### MAGE-A4 promotes PCNA mono-ubiquitination and TLS

**Figure 7:**
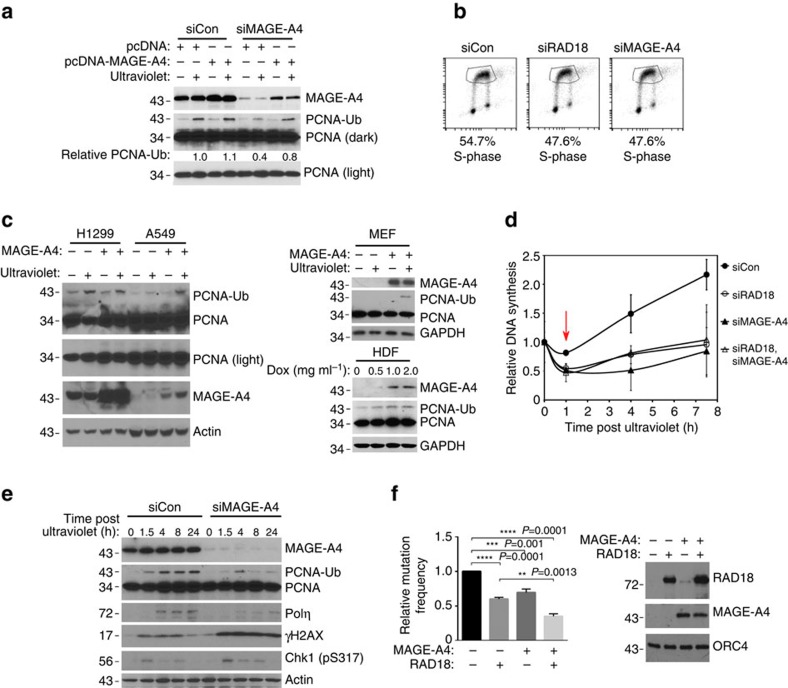
MAGE-A4 promotes TLS and DNA-damage tolerance. (**a**) H1299 cells were transiently transfected with MAGE-A4 or non-targeting siRNAs. After 16 h, cells were transfected with a siRNA-resistant MAGE-A4 expression plasmid (or empty vector control). Forty-eight hours later, cells were sham or ultraviolet irradiated (20 J m^−2^) and harvested for immunoblot analysis after 2 h. (**b**) H1299 cells were transfected with siRNA against RAD18, MAGE-A4 or non-targeting siRNA. Forty-eight hours later, cells were pulsed labelled with BrdU (10 μM) for 1 h and collected for flow cytometry. (**c**) H1299, A549 or mouse embryonic fibroblast (MEF) cells were transfected with a MAGE-A4 expression plasmid or empty vector. After 48 h, cells were sham or ultraviolet irradiated (20 J m^−2^) and extracted 2 h later for immunoblotting. Human dermal fibroblasts (HDFs) stably transduced with a pINDUCER-MAGE-A4 were treated with indicated doxycycline concentrations for 48 h and then collected for immunoblotting. (**d**) H1299 cells were transfected with siRNA against RAD18 and MAGE-A4 (or with non-targeting oligonucleotides). Twenty-four hours post transfection, cells were re-plated in 24-well dishes and ultraviolet irradiated (5 J m^−2^) 48 h later. DNA synthesis rates were measured immediately before and at different times after ultraviolet treatment. (**e**) H1299 cells were transfected with siRNA against MAGE-A4 or with non-targeting siRNA. Seventy-two hours post transfection, cells were sham or ultraviolet irradiated (5 J m^−2^) and harvested at different times for immunoblotting. (**f**) 293T cells were co-transfected with ultraviolet-damaged pSP189 reporter plasmid and MAGE-A4 or RAD18 expression vectors. Forty-eight hours later, 293T cell extracts were collected for immunoblot analysis of MAGE-A4 and RAD18 (right). Recovered pSP189 plasmid was transformed into electro-competent MBM7070 bacteria and pSP189 mutation rates were determined by enumerating blue and white bacterial colonies. Data represent means±s.e.m. of four independent experiments each performed in triplicate. *P*-values were calculated using a two-tailed Student's *t*-test. Baseline mutation rates for the experiments ranged from 5.6 to 9.6%.

Increased expression of RAD18 can substantially enhance both basal and genotoxin-induced PCNA mono-ubiquitination^5^. Therefore, we determined whether MAGE-A4 contributes to RAD18-dependent TLS pathway activation in cancer cells. As shown in Fig. 7a and Supplementary Fig. 6, siRNA-mediated MAGE-A4 knockdown in H1299 cells led to an attenuation of ultraviolet-inducible PCNA mono-ubiquitination. The reduced PCNA ubiquitination of MAGE-depleted cells was rescued by co-transfection of siRNA-resistant MAGE-A4 (Fig. 7a). 5-Bromodeoxyuridine (BrdU) labelling and fluorescence-activated cell sorting analyses revealed no effect of MAGE-A4 depletion on DNA synthesis or cell cycle parameters (Fig. 7b). Therefore, the reduced PCNA mono-ubiquitination of MAGE-A4-depleted cells was not secondary to cell cycle changes.

As MAGE-A4 depletion led to reduced PCNA mono-ubiquitination in H1299 cells, we also asked whether forced expression of MAGE-A4 in cells lacking the protein endogenously was sufficient to induce PCNA mono-ubiquitination. As shown in Fig. 7c, ectopic overexpression of MAGE-A4 in A549 cells enhanced PCNA mono-ubiquitination in response to low ultraviolet doses. Overexpressed MAGE-A4 did not affect PCNA mono-ubiquitination in H1299 cells (which already express high levels of endogenous MAGE-A4). MAGE-A4 also induced PCNA mono-ubiquitination when ectopically expressed in non-transformed mouse embryonic fibroblasts and human dermal fibroblasts. MAGE-A4 expression did not induce PCNA mono-ubiquitination in *RAD18*^*−/−*^ cells, demonstrating that the stimulatory effect of MAGE-A4 on PCNA mono-ubiquitination was RAD18 dependent.

As MAGE-A4 promotes RAD18-mediated PCNA mono-ubiquitination (Fig. 7a–c), we determined the potential contribution of MAGE-A4 to replication of damaged DNA. RAD18-depleted cells fail to recover appropriately from DNA damage-induced inhibition of DNA synthesis^5^. Interestingly, MAGE-A4 depletion partially phenocopied the defective S-phase recovery of RAD18-depleted H1299 cells from ultraviolet-induced replication arrest (Fig. 7d). Moreover, co-depletion of RAD18 and MAGE-A4 did not have additive inhibitory effects on S-phase recovery after ultraviolet treatment (Fig. 7d). Similar to phenotypes described in RAD18-depleted cells, the defective recovery of MAGE-A4-depleted cells from S-phase arrest was associated with persistence of γH2AX (Fig. 7e). RAD18 expression was also MAGE-A4 dependent in H157 and H650 adenocarcinoma cells and in U2OS osteosarcoma cells (which express endogenous MAGE-A4; see Supplementary Fig. 7a,b). Similar to H1299 cells, MAGE-A4 depletion led to an attenuation of PCNA mono-ubiquitination and increased γH2AX after ultraviolet treatment in U2OS cells (Supplementary Fig. 7c). Taken together, the results of Fig. 7c–e indicate a role for MAGE-A4 in facilitating TLS and recovery from DNA damage-induced replication fork stalling.

To determine whether MAGE-A4 impacts RAD18-mediated genome maintenance we used an established assay in which RAD18 promotes error-free bypass of an ultraviolet-damaged pSP189 reporter plasmid, thereby suppressing mutagenesis^38^. As shown in Fig. 7f, ectopic expression of RAD18 in 293T cells suppressed mutagenesis of the ultraviolet-damaged supF reporter by 40%, consistent with previous reports^38^. Interestingly, MAGE-A4 expression alone led to a 31% decrease in mutagenesis. When co-expressed with RAD18, MAGE-A4 further enhanced the suppressive effect of RAD18 on mutagenesis. As expected, MAGE-A4 induced the expression of endogenous and ectopically co-expressed RAD18 coincident with suppression of mutagenesis (Fig. 7f). MAGE-A4 overexpression did not affect DNA synthesis rates or ultraviolet-checkpoint recovery of 293T cells (Supplementary Fig. 8). Therefore, MAGE-A4 can specifically influence replicative bypass of ultraviolet-induced DNA lesions, further consistent with its novel role in regulating RAD18 levels and TLS activity in cancer cells.

## Discussion

Potts and colleagues^25^ made the seminal discovery that many MAGE proteins bind and activate E3 ubiquitin ligases, contributing to deregulated ubiquitin signalling in cancer cells. Our work identifies RAD18 as a target of MAGE-A4 and provides a new potential mechanism by which genome maintenance and genome stability can be altered in cancer cells.

There are interesting similarities and differences in the relationship between MAGE-A4 and RAD18 when compared with previously described MAGE-E3 ligase associations. For example, the conserved di-leucine motif required by other MAGE family members to activate their cognate E3 ligases^25^ is also necessary for MAGE-A4 to stabilize RAD18. However, although other MAGEs are allosteric activators of their associated E3 ligases^25^, MAGE-A4 does not stimulate catalytic activity of purified recombinant RAD18 under defined *in vitro* conditions. Instead MAGE-A4 stabilizes RAD18 to confer increased PCNA mono-ubiquitination and TLS. Therefore, this study provides a new paradigm for MAGE-induced reprograming of ubiquitin signalling via altered E3 ligase stability in cancer cells.

It is possible that MAGE-A4–RAD18 signalling also occurs during normal mammalian development and in non-pathological situations. Similar to MAGE proteins, Rad18 is expressed at high levels in germ cells and male *rad18*^*−/−*^mice have impaired spermatogenesis and fertility^39^. However, in preliminary experiments we have not detected Mage-a4 (or other Mage proteins) in anti-RAD18 immunoprecipitates from mouse testes extracts. Therefore, we favour the hypothesis that RAD18 binding is a ‘neomorphic' activity of aberrantly expressed MAGE-A4 in cancer cells.

Remarkably, although several MAGE-E3 ubiquitin ligase complexes have been characterized^25^, no conserved sequence motifs (on MAGE-A4 family member or E3 ligases) mediate these protein–protein associations. Thus, the mechanism of association appears to be different for every MAGE-E3 ligase complex. Our structure–function analyses show that MAGE-A4 binds and stabilizes RAD18 via the RAD6-binding domain. Reportedly, p95/NBS1 also associates with the RAD6-binding domain of RAD18^34^. Physiologically, RAD18 exists as an asymmetric hetero-trimer comprising two RAD18 molecules in complex with one molecule of RAD6 (ref. 40). Therefore, we hypothesize that one RAD18 molecule in the [RAD18]_2_–RAD6 heterotrimer has a ‘free' RAD6-binding domain that is available to interface with MAGE-A4, p95 and perhaps additional proteins. This hypothesis predicts that MAGE-A4 and p95 (or other proteins) may compete for RAD18 binding in cancer cells, and that such competition may have an impact on genome maintenance events involving RAD18–p95 associations. MAGE-A4 lacks the RAD6-like β-sheet and therefore interacts with RAD18 via a distinct mechanism. Clearly, biophysical and crystallographic studies will be necessary to fully characterize the putative [RAD18]_2_–RAD6–MAGE-A4 complex that exists in cancer cells.

The only other documented E3 ligase-binding partner of MAGE-A4 is TRIM69 and the mechanism of MAGE-A4–TRIM69 association has not been studied. Other known effectors of MAGE-A4 are the transcription factor Miz1 (ref. 37) and the liver oncoprotein gankyrin^36^, which both bind a C-terminal region of MAGE-A4. We show here that the minimal MAGE-A4 C-terminal region (AB) that regulates Miz1 and gankyrin is insufficient to stabilize RAD18. Indeed, none of the major conserved MAGE-A4 domains retain RAD18-stabilizing activity in isolation. Therefore, RAD18 binding is probably not a modular interaction mediated by individual MAGE-A4 domains. Instead, the overall tertiary structure adopted by MAGE-A4 is likely to be involved in the formation of the MAGE-A4–[RAD18]_2_–RAD6 complex. The finding that all MAGE-A4 mutants failed to stabilize RAD18 may further support the idea that multiple regions of the MAGE-A4 are required for its RAD18 association. Other MAGE family members with a MAGE-A4-related domain organization do not share RAD18-stabilizing activity, further suggesting that unique or specific tertiary structural determinants are required for MAGE-A4 to bind and stabilize RAD18.

Regardless of the mechanism of MAGE-A4–RAD18 interaction, we show here that endogenous MAGE-A4 confers RAD18 stability and expression in cancer cells. TLS is generally assumed to be a housekeeping genome maintenance mechanism and it has not been suggested that expression or activities of core TLS pathway components are significantly different between cell types. However, expression levels of RAD18 and other TLS proteins (including Polη, Polι and PCNA) vary greatly between different cultured cell lines (Supplementary Fig. 9). What then are the possible consequences of variable RAD18 and TLS polymerase expression on genome stability and carcinogenesis? RAD18-deficient cells do not recruit TLS polymerases to replication forks^5^,^15^ and exhibit reduced lesion bypass activity^17^. Conversely, RAD18 overexpression stimulates PCNA ubiquitination, recruits Y-family polymerases to replication forks, promoting TLS^5^,^41^. Therefore, the repertoire of Y-family DNA polymerases and the degree to which different TLS polymerases respond to RAD18 and PCNA mono-ubiquitination may have enormous impact on genome stability when RAD18 is present at aberrantly high levels. For example, HeLa cells express unusually high levels of Polι compared with H1299 cells (Supplementary Fig. 9). Polι has exceptionally low fidelity, misincorporating dGTP more frequently than the correct dATP across ‘T' on undamaged templates^42^. Therefore, increased RAD18 expression in a cell with aberrantly high Polι levels cell will probably have a severe effect on replication fidelity. Polκ overexpression in cultured cells leads to insertions and deletions^43^. Consequently, Polκ activation in response to aberrant RAD18 overexpression might cause elevated frequency of indel mutations. Moreover, TLS polymerases have low processivity compared with replicative DNA polymerases. Therefore, elevated RAD18 expression and PCNA mono-ubiquitination could lead to rampant recruitment of Y-family polymerases to undamaged DNA, causing replication fork slowdown and/or other defects that result in ‘fork collapse' and compromise genome stability due to DSB formation. A potential role for MAGE-A4–RAD18 as a mutagenic driver or source of genomic instability in cancer cells owing to inappropriate TLS polymerase activation is highly likely.

Maiorano and colleagues^41^ recently showed that ectopic RAD18 overexpression can lead to DNA damage tolerance. Potentially, MAGE-A4-induced RAD18 expression might contribute to tumorigenesis by enhancing DNA-damage tolerance via TLS (and perhaps additional RAD18-mediated DNA repair pathways such as homologous recombination^44^ and cross-link repair^28^). Neoplastic cells must endure endogenous stresses including ROS-induced DNA damage and other forms of DNA replication stress^45^. Collectively, TLS polymerases can perform bypass of oxidative lesions (such as 8-oxo-dG and AP sites) potentially conferring tolerance of oncogene-induced ROS. In addition, TLS polymerases can facilitate ongoing DNA synthesis in cells undergoing oncogene-induced re-replication^22^ (one of the earliest responses to oncogene activation in untransformed cells^46^). Therefore, increased TLS capacity afforded by MAGE-A4–RAD18 may contribute to tolerance of spontaneously arising DNA damage and replication stress, thereby facilitating neoplastic cell survival and tumour progression.

Clearly, future experiments are necessary to determine the potential contribution of MAGE-A4 and RAD18 to genome destabilization and tolerance of oncogenic stress. In addition to promoting tolerance of intrinsic oncogene-induced sources of stress (such as ROS and re-replication), RAD18 confers tolerance of chemo/radiotherapy^47^,^48^. Therefore, the MAGE-A4–RAD18 signalling axis may represent an attractive druggable target whose inhibition is innocuous to normal cells but selectively sensitizes cancer cells to intrinsic and therapy-induced DNA damage and replication stress.

## Methods

### Cell culture and transfection

hTERT-expressing human dermal fibroblasts were provided by Dr William Kaufmann (UNC Chapel Hill). Primary mouse embryonic fibroblasts were derived from E13.5 embryos of WT C57/BL6 mice. Cancer cell lines H1299, A549, HeLa, U2OS, H157, H650, HCT116 and 293T were purchased from the American Type Culture Collection (ATCC) and used for the described experiments without further authentication. It is noteworthy that the H157 squamous cell lung carcinoma cell line is on the International Cell Line Authentication Committee (ICLAC) misidentified cell list. According to the ATCC, H157 is identical to the H1264 squamous cell lung carcinoma cell line. In the experiments shown in Supplementary Fig. 7, H157 cells were used solely as one (of several) example of independent cancer cell lines in which RAD18 expression is MAGE-A4 dependent. All cell lines tested negative for mycoplasma contamination using the ATCC Universal Mycoplasma Detection Kit (ATCC 301012K). All cell lines were cultured in DMEM medium supplemented with 10% fetal bovine serum and penicillin–streptomycin (1%). Plasmid DNA and siRNA oligonucleotides were transfected using Lipofectamine 2000 (Invitrogen) according to the manufacturer's instructions, except that concentrations of plasmid DNA and Lipofectamine 2000 were used in each transfection reaction were decreased by 50% to reduce toxicity.

### Adenovirus construction and infection

Adenovirus construction, purification and infections were performed as described previously^27^,^49^. H1299 cells were typically infected with 0.1−1.0 × 10^9^ pfu ml^−1^ and titrated to achieve near-endogenous expression levels of RAD18 and other proteins.

### Expression plasmids

GST-RAD18, GST-RAD6 and GST-MAGE-A4 were expressed using the pGEX2T vector (GE Healthcare) and purified from BL21 (DE3) *Escherichia coli* (Invitrogen) as described previously^27^. Hexa-histidine-tagged MAGE-A4 was expressed using the pRSET vector (Invitrogen V351-20) and purified from BL21 (DE3) *E. coli* bacteria. Mammalian expression vectors for HA- and MYC-tagged forms of RAD18 have been described previously^26^,^27^. To generate MAGE-A4 expression vectors, the MAGE-A4 open reading frame was PCR amplified from H1299 genomic DNA and subcloned into the pcDNA3.1(−) expression plasmid. MAGE-A4 mutants harbouring internal deletions and individual nucleotide substitutions were derived by PCR using conventional methods. The primers used to make MAGE-A4 mutants are: 5′-F WT (5′- CGCGGATCCGCCACCATGTCTTCTGAGCAGAAGAGTCAGCAC -3′), 3′-R WT (5′- AACAAGCTTTCAGACTCCCTCTTCCTCCTCTAACAAAG -3′); 5′-F HelixB (5′- CGCGGATCCGCCACCATGGATGGCCTGCTGGGTAATAATCAG -3′), 5′-F HelixA+B (5′- CGCGGATCCGCCACCATGTCCTTGTTCCGAGAAGCACTCAGTAAC -3′); ΔWHA-F (5′- GCCTTTCCTATGGTCCAAGGGC -3′), ΔWHA-R (5′- GCCCTTGGACCATAGGAAAGGC -3′); ΔWHA-F (5′- TGACGCAGAGGATGGCCTGC -3′), ΔWHA-R (5′- GCAGGCCATCCTCTGCGTCA -3′); ΔWHB-F (5′- GCCTTTCCTATGGTCCAAGGGC -3′), ΔWHB-R (5′- GCCCTTGGACCATAGGAAAGGC -3′); ΔMage-F (5′- GACGCAGAGGGTCCAAGGGC -3′), ΔMage-R (5′- GCCCTTGGACCCTCTGCGTC -3′); L121/2A-F (5′- CTCATTTTGCGGCCCGCAAG -3′), L121/2A-R (5′- CTTGCGGGCCGCAAAATGAG -3′); S90A-F (5′- GTTCCAGCGCCCAAGAAGAGG -3′), S90A-R (5′- CCTCTTCTTGGGCGCTGGAAC -3′); and S90D-F (5′- GGGTTCCAGCGATCAAGAAGAGG -3′), S90D-R (5′- CCTCTTCTTGATCGCTGGAACCC -3′). The identities of all complementary DNA inserts were confirmed by sequencing. MYC–TRIM69 was a gift from Dr Angelique Whitehurst (UT Southwestern) and expression plasmids encoding FLAG-tagged MAGE-A4, MAGE-A12, MAGE-B10 and MAGE-A1 were obtained from the UNC Tissue Culture Core Facility Orfeome collection.

### RNA interference

siRNAs were incubated with Lipofectamine 2000 and serum-free Optimem for 15 min at room temperature in the dark. Cells were then trypsinized and resuspended in 1 ml of medium and plated directly into the siRNA/Optimem/Lipofectamine solution at 50% confluence and incubated for 72 h. Sequences of siRNA oligonucleotides used here are as follows: control non-targeting siRNA, 5′- UAGCGACUAAACACAUCAA -3′ (Thermo Fisher Scientific); RAD18 3′-untranslated region siRNA, 5′- UUAUAAAUGCCCAAGGAAAUU -3′; MAGE-A4 siRNA #1, 5′- AGUGUGAAUUCACCGUGAA -3′, MAGE-A4 siRNA #2 (targeting the 3′-untranslated region), 5′- GUGAAAUAGGUGAGAUAAAUU -3′; and USP7, 5′- AAGCGUCCCUUUAGCAUUAUU -3′. For MAGE-A4 depletions, siRNA#1 was used unless otherwise indicated.

### Genotoxin treatment

For ultraviolet C (UVC) treatment, growth medium was removed from cultured cells and replaced with PBS. The resulting culture dishes plates were irradiated using an ultraviolet cross-linker (Stratagene) or left untreated for control. The UVC dose delivered to the cells was confirmed with an ultraviolet radiometer (UVP, Inc.). Following ultraviolet or sham irradiation, cells were re-fed with complete growth medium and returned to the incubator. For CPT treatments, cells were treated with 2 μM CPT and incubated for 2 h.

### Fluorescence microscopy

H1299 cells were grown to ∼60% confluency on glass-bottom plates (Mat-tek) and then transfected with a CFP-RAD18-WT expression plasmid. Twenty hours after transfection, cells were ultraviolet irradiated (20 J m^−2^) or sham treated and fixed 6 h later for staining with anti-MAGE-A4 and fixed-cell imaging on a Zeiss 710 confocal microscope, in the UNC Microscopy Services Laboratory core facility, as described previously^30^.

### Immunoprecipitation and immunoblotting

To prepare extracts containing soluble and chromatin-associated proteins, monolayers of cultured cells typically in 60 mm plates were washed three times in ice-cold PBS and lysed in 500 μl of ice-cold cytoskeleton buffer (CSK buffer; 10 mM Pipes pH 6.8, 100 mM NaCl, 300 mM sucrose, 3 mM MgCl_2_, 1 mM EGTA, 1 mM dithiothreitol, 0.1 mM ATP, 1 mM Na_3_VO_4_, 10 mM NaF and 0.1% Triton X-100) freshly supplemented with Protease Inhibitor Cocktail and Phostop (Roche). Lysates were centrifuged at 1,000 *g* for 2 min, to remove the CSK-insoluble nuclei. Supernatants were removed and further centrifuged at 10,000 *g* for 10 min, to obtain a clarified fraction containing a mixture of cytosolic plus nucleosolic proteins. The detergent-insoluble nuclear fractions were washed once with 1 ml of CSK buffer and then resuspended in a minimal volume of CSK before analysis by SDS–PAGE and immunoblotting.

For all immunoprecipitation experiments, input samples were normalized for protein concentration. Magnetic beads containing covalently conjugated antibodies against epitope tags were added to the extracts and incubations were performed overnight at 4 °C using rotating racks.

Immune complexes were recovered using magnetic stands. The beads were washed five times with 1 ml CSK (5–10 min per wash), to remove nonspecifically associated proteins. The washed immune complexes were boiled in protein loading buffer for 10 min, to release and denature for SDS–PAGE.

For immunoblotting, cell extracts or immunoprecipitates were separated by SDS-PAGE, transferred to nitrocellulose membranes, and incubated overnight with the following primary antibodies: PCNA (sc-56), Chk1 (sc-7898), β-actin (sc-130656), cyclin E (sc-198), GAPDH (sc-32233), MAGE-A4 (sc-292429), Pan-MAGE-A (sc71537) and GST (sc-53909) from Santa Cruz Biotech (Santa Cruz, CA); Polη (A301-231A), Polι (A301-304A), RAD6 (A300-281A), RAD18 (A301-340A) and USP7 (A300-033A) from Bethyl Laboratories (Montgomery, TX); p42 MAPK (9107) and MYC-Tag (2276) from Cell Signaling; γH2AX (05-636) from Millipore; and Cdc45 rat monoclonal antibody as previously described^50^. Antibody dilutions used for immunoblotting were 1:1,000, with exceptions for the following antibodies: PCNA (1:500), GAPDH (1:2,000) and ΥH2AX (1:2,000). Uncropped images of the most important western blottings are shown in Supplementary Fig. 10.

### *In vitro* protein-binding assays with lysate

Mammalian cells were transfected with 2 μg of plasmid and incubated for 48 h. Cell lysate was collected in CSK buffer and centrifuged at 13,300 r.p.m. to clear lysate. Recombinant GST–RAD18 fragments (100 ng) were incubated in 1 ml CSK with 100 μg cleared lysate for 2 h at 4 °C. Fifty microlitres of Glutathione sepharose beads (GE Healthcare 17-0756-01) was added to the solution and incubated for 2 h more at 4 °C. Beads and complexes were collected by centrifugation and washed three times in CSK+1% BSA, then resuspended in water and 4 × Laemmli buffer and boiled for 10 min.

### *In vitro* RAD18–MAGE-A4 recombinant protein binding assay

Recombinant 6 × His–MAGE-A4 (1 μg) was incubated in 1 ml of CSK+1% BSA with either GST or GST–RAD18 (0.3 μg) for 2 h at 4 °C. Fifty microlitres of Glutathione sepharose beads (GE Healthcare 17-0756-01) was added to the solution and incubated for 2 h more at 4 °C. Beads and complexes were collected by centrifugation and washed three times in CSK+1% BSA, then resuspended in water and 4 × Laemmli buffer and boiled for 10 min.

### *In vitro* degradation of RAD18

HA–RAD18 was expressed alone or in combination with MAGE-A4 in 293T cells. Cells were collected using CSK buffer. HA–RAD18 complexes were isolated by immunoprecipitation using anti-HA magnetic beads (MBL Intl M-1329) for 2 h at 4 °C. Beads were washed with CSK and incubated for 1 h at 37 °C in 2 mM MgCl_2_, 1 mM creatine phosphate, 25 U ml^−1^ creatine phosphokinase (FisherSci, ICN10050990), PBS and 1 mg ml^−1^ of rabbit reticulocyte lysate, untreated (L4151), from Promega, as a source of ubiquitination factors and proteasome activity, as described by Hernandez-Pigeon *et al*.^51^.

### Flow cytometry

Cells were labelled with 10 μM BrdU immediately before harvest. Cells were collected by trypsinization, fixed in 35% ethanol for 24 h, then stained with anti-BrdU and propidium iodide as previously described^27^. Stained nuclei were analysed by flow cytometry on an Accuri C6 flow cytometer (BD, Oxford, UK) using the manufacturer's software.

### *In vitro* PCNA ubiquitination assay

Recombinant RAD18–RAD6 complex was purified from baculovirus-infected Sf9 cells and incubated with recombinant PCNA in the presence of E1, ubiquitin and an ATP-regenerating system as described previously^52^.

### SupF mutagenesis assay

293T cells were co-transfected with a ultraviolet-irradiated (500 J m^−2^) pSP189 reporter plasmid^53^ and control, RAD18 or MAGE-A4 expression vectors using Lipofectamine 2000. Forty-eight hours later, pSP189 was recovered from the 293T cells using a DNA miniprep kit (Qiagen, Hilden, Germany). Purified plasmid DNA was DpnI digested and electroporated into the MBM7070 bacterial strain. The mutation frequency in the supF coding region was determined by enumerating the ratios of blue (WT) and white (mutant) colonies.

### Mass spectrometry

PBS-washed cell pellets from HA-RAD18-expressing (and control) cells were lysed with CSK and digested with 1,000 U ml^−1^ of RNase-free DNase I (Roche) at 25 °C for 30 min. The resulting mixtures were sonicated to dissociate the nuclei. Insoluble material was removed by centrifugation at 10,000 *g* for 10 min. The resulting supernatant (containing cytosol, nucleosol and solubilized chromatin proteins) was used for immunoprecipitation of RAD18 complexes.

Anti-HA-conjugated magnetic beads (MBL Intl, M-1329) were incubated with HA–RAD18-containing supernatant for 4 °C for 3 h. Following incubation, beads were washed in CSK. The protein complexes were digested directly off of the beads using FASP Protein Digestion Kit (Protein Discovery #44250).

Peptides were separated by reversed-phase nano-high-performance liquid chromatography with a nanoAquity UPLC system (Waters Corp.). Peptides were first trapped in a 2-cm trapping column (75-μm inside diameter (ID), Michom Magic C18 beads of 5.0-μm particle size, 200-Å pore size) and then separated on a self-packed 25-cm column (75-μm ID, Michom Magic C18 beads of 5.0-μm particle size, 100-Å pore size) at room temperature. The flow rate was 350 nl min^−1^ over a gradient of 1% buffer B (0.1% formic acid in acetonitrile) to 30% buffer B in 200 min. Next, a following wash raised buffer B to 70%. The identity of the eluted peptides was determined with an in-line LTQ-Orbitrap Velos mass spectrometer (Thermo Scientific). The ion source was operated at 2.0–2.4 kV with the ion transfer tube temperature set at 250 °C. Full MS scan (300 to 2,000 *m/z*) was acquired in Orbitrap at 60,000 resolution setting; data-dependent MS2 spectra were acquired in LTQ by collision-induced dissociation with the 15 most intense ions. Precursor ions were selected on the basis of charge states (2 or 3) and intensity thresholds (above 5,000) from the full scan; dynamic exclusion (one repeat every 30 s, with a 60-s exclusion time window) was also taken into account. The polysiloxane lock mass of 445.120030 was used throughout spectral acquisition.

*Protein identification, quantification and filtering*. Raw data were analysed using Sorcerer-SEQUEST (build 5.1.1, SageN Research) and the Transproteomic Pipeline (TPP v4.7.1). MS/MS spectra were searched against the human UniProtKB/Swiss-Prot sequence database (downloaded February 2015) supplemented with common contaminants, that is, porcine (Swiss-Prot P00761) and bovine (P00760) trypsin, and further concatenated with its reversed copy as a decoy. Search parameters used were a precursor mass between 400 and 4,500 amu, up to 2 missed cleavages, precursor-ion tolerance of 3 amu, accurate mass binning within PeptideProphet, semi-tryptic digestion, a static carbamidomethyl cysteine modification and variable methionine oxidation. False discovery rates were determined by ProteinProphet and minimum protein probability cutoffs resulting in a 1% false discovery rate were selected individually for each experiment. The resulting spectral count data from controls and HA–RAD18-WT APMS experiment were input into the Spotlite web application using SAINTexpress (version 3.1.0), to determine protein–protein interaction probabilities by modelling the expected spectral count distribution of true and false interactions. In addition, raw data were re-searched and signal intensity was quantified using the MaxQuant LFQ algorithm with the identical sequence database and search parameters, except a 20-p.p.m. precursor mass tolerance, fully tryptic digestion and match between runs were used.

### Data availability

The authors declare that the data supporting the findings of this study are available within the article and its Supplementary Information files.

## Additional information

**How to cite this article:** Gao, Y. *et al*. A neomorphic cancer cell-specific role of MAGE-A4 in *trans*-lesion synthesis. *Nat. Commun.* 7:12105 doi: 10.1038/ncomms12105 (2016).

## Supplementary Material

Supplementary Information Supplementary Figures 1-10, Supplementary Note 1 and Supplementary References

Supplementary Data 1 AD18 Mass Spec SAINT Analysis
