# Supplementary material for: A neomorphic cancer cell-specific role of MAGE-A4 in trans-lesion synthesis
Source: Nat Commun. 2016 Jul 5;7:12105. doi: 10.1038/ncomms12105 (PMC4935975; doi:10.1038/ncomms12105)
Supplement: Supplementary Information — Supplementary Figures 1-10, Supplementary Note 1 and Supplementary References [file ncomms12105-s1.pdf]

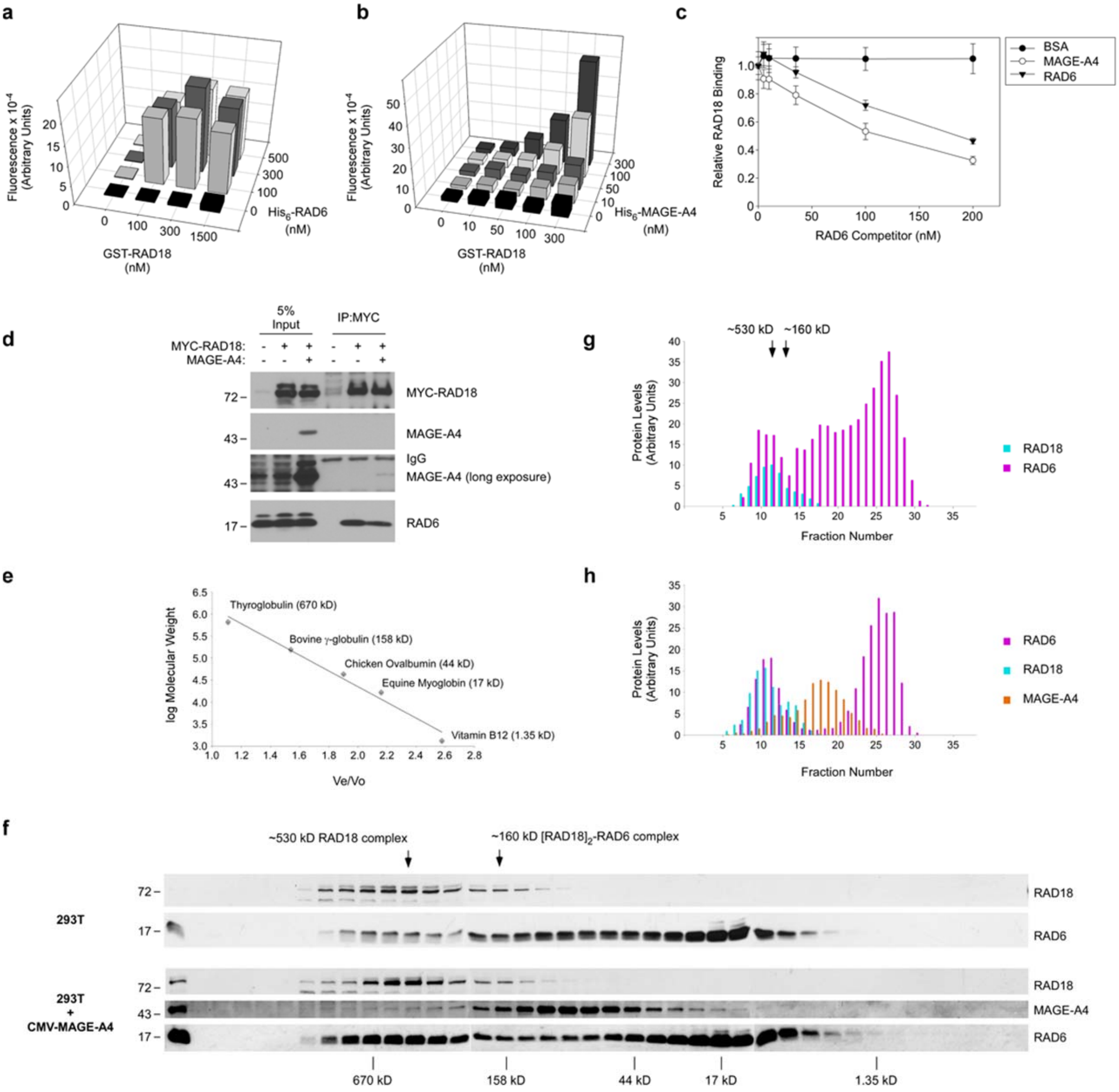


**Supplementary Fig. 1 RAD6 and MAGE-A4 associate with the RAD6-binding domain of RAD18.**

(a) Amplified Luminescent Proximity Homogeneous Assay (ALPHA) screen assay showing association of RAD18 and RAD6. The indicated concentrations of purified recombinant GST-RAD18 335-400 and His-tagged full-length RAD6 were incubated in 384-well plates (16 replicate wells for each experimental condition) in a reaction volume of 6 µl for 1 h at room temperature in the dark. Next, 3 µl of nickel chelate acceptor beads (Perkin Elmer 6760619C)

were added to give a concentration of 20 μg/ml and incubations were continued for 1 h. Finally 3 μl of glutathione donor beads (Perkin Elmer 6765300) were added (20 μg/ml final concentration) and incubations were continued for one more hour. Plates were analyzed using the Perkin Elmer Envision plate reader to excite at 680 nm and detect fluorescence at 520 nm.

(b) The ALPHAscreen assay was used to detect the association of GST-RAD18 335-400 with His-tagged full-length MAGE-A4, exactly as described for RAD18-RAD6 association in (a) above. Note that the binding curves in (a) and (b) cannot be compared directly because in ALPHAscreen assays the magnitude of signal for each unique protein-protein interaction is determined by specific protein conformations and tag proximities.

(c) ALPHAscreen assay was used to detect competition between RAD6 and MAGE-A4 for RAD18-binding. In separate reactions His-RAD6 (25 nM) or His-MAGE-A4 (200 nM) were incubated with equimolar GST-RAD18 in the presence of different concentrations (0-200 nM) of untagged competitor RAD6 (or BSA for control). Donor and acceptor beads were added to the reactions and plates were analyzed as described in (a) above. To plot the data, the effect of untagged RAD6 (or BSA) on fluorescence was normalized to emission signals obtained in the RAD18/His-RAD6 and RAD18/His-MAGE-A4 reactions without added protein. Error bars represent the mean of 16 replicate wells +/- SEM for each experimental condition.

(d) Effect of MAGE-A4 expression on RAD18-associated RAD6 in cultured cells. 293T cells were transfected with expression vectors encoding MYC-RAD18 and MAGE-A4 (or empty vector for control). Cell lysates were prepared 48 h after transfection, and RAD18 was immunoprecipitated using anti-MYC beads. Levels of RAD18-associated RAD6 were determined by SDS-PAGE and immunoblotting. Note that in this experiment, levels of ectopically-expressed RAD18 were very high and no longer sensitive to co-expressed MAGE-A4.

(e)-(h) Size fractionation of RAD18, RAD6 and MAGE-A4 complexes in cultured cells. Exponentially-growing cultures of 293T cells were transfected with empty vector, CMV-GFP, or with a CMV-MAGE-A4 expression plasmid. 48 h later GFP fluorescence was used to confirm efficient (~90%) transfection efficiency. Cells were lysed in 300 μl of CSK without sucrose and supplemented with 1 μg/ml (25,000 units/ml) of Benzonase. Lysates were incubated at room temperature for 15 minutes to digest chromatin, then centrifuged at 21,000g for 20 min. 250 μl of each clarified cell lysate (~2.0 mg) was loaded onto a 25ml Sephadex 200 gel filtration column that was calibrated with appropriate molecular weight markers (e). The column was eluted with sucrose-free CSK. 0.5 ml fractions were collected and 30 μl of each fraction was analyzed by SDS-PAGE and immunoblotting with antibodies against RAD18, RAD6 and MAGE-A4 (f). Relative levels of RAD18, RAD6 and MAGE-A4 between different fractions were determined by densitometry and are plotted in panels (g) and (h). The fractions containing the ~160 kD $[RAD18]_2$-RAD6 complex predicted by biophysical studies and a previously undescribed ~530 kD RAD18 and RAD6-containing complex are indicated.

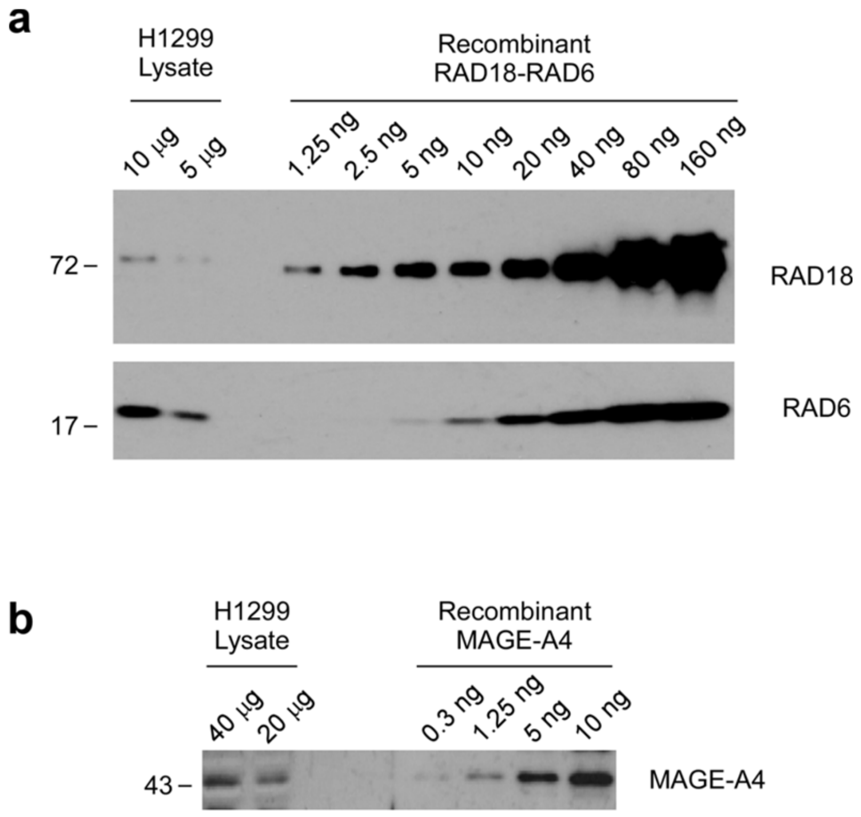


**Supplementary Fig. 2 Quantitative immunoblot analysis of RAD18, RAD6 and MAGE-A4 expression levels in H1299 cells.**

The indicated amounts of H1299 whole cell lysate, recombinant RAD18-RAD6 (a), and MAGE-A4 (b) proteins were analyzed by SDS-PAGE and immunoblotting. By comparing expression levels of proteins in cell lysates with defined quantities of purified protein standards we estimate that 1 µg of H1299 cell lysate contains 41 pg (0.74 fmol) of RAD18, 719 pg (42 fmol) of RAD6, and 88 pg (2.5 fmol) of MAGE-A4. Therefore, the RAD18:RAD6:MAGE-A4 stoichiometry in H1299 cells is approximately 1:57:3.

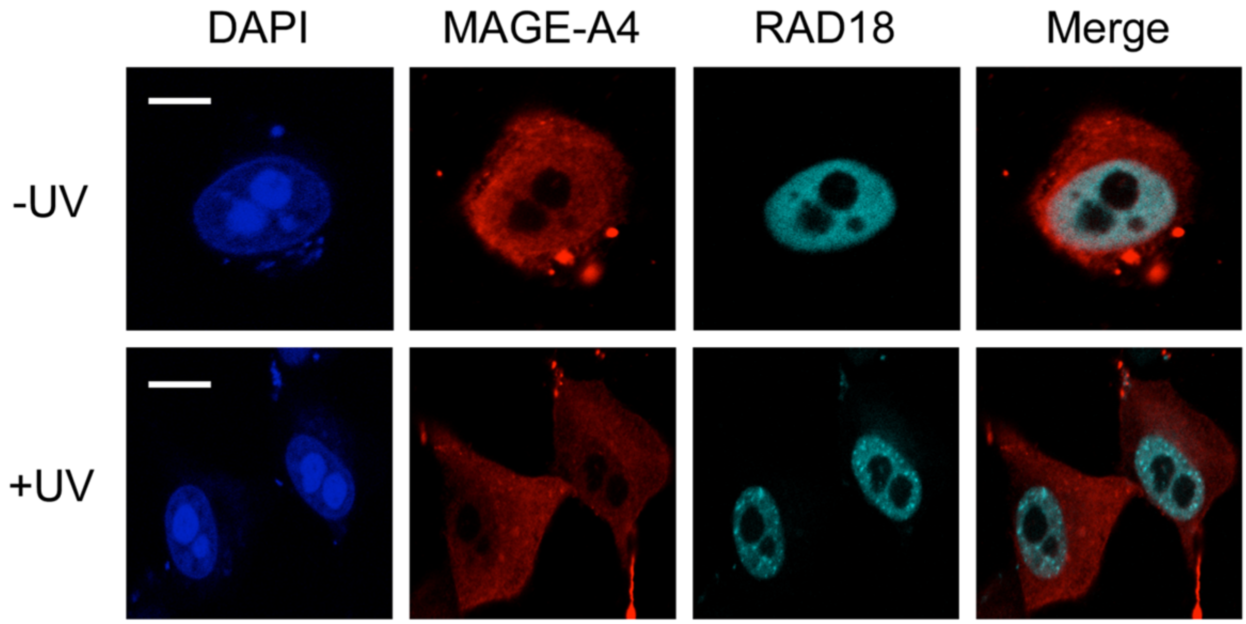


**Supplementary Fig. 3  Expanded version of Fig. 3c showing UV-induced distribution of RAD18 but not of MAGE-A4 to nuclear foci.**

H1299 cells were transiently transfected with an expression plasmid encoding CFP-RAD18 or with an empty vector for control.  Transfected cells were UV-irradiated (20 J/$m^2$) or were left untreated.  After 6 h, cells were fixed and stained with antibodies against MAGE-A4.  The subcellular distribution of MAGE-A4 and CFP-RAD18 was visualized using immunofluorescence microscopy.  Scalebar = 10 μm.

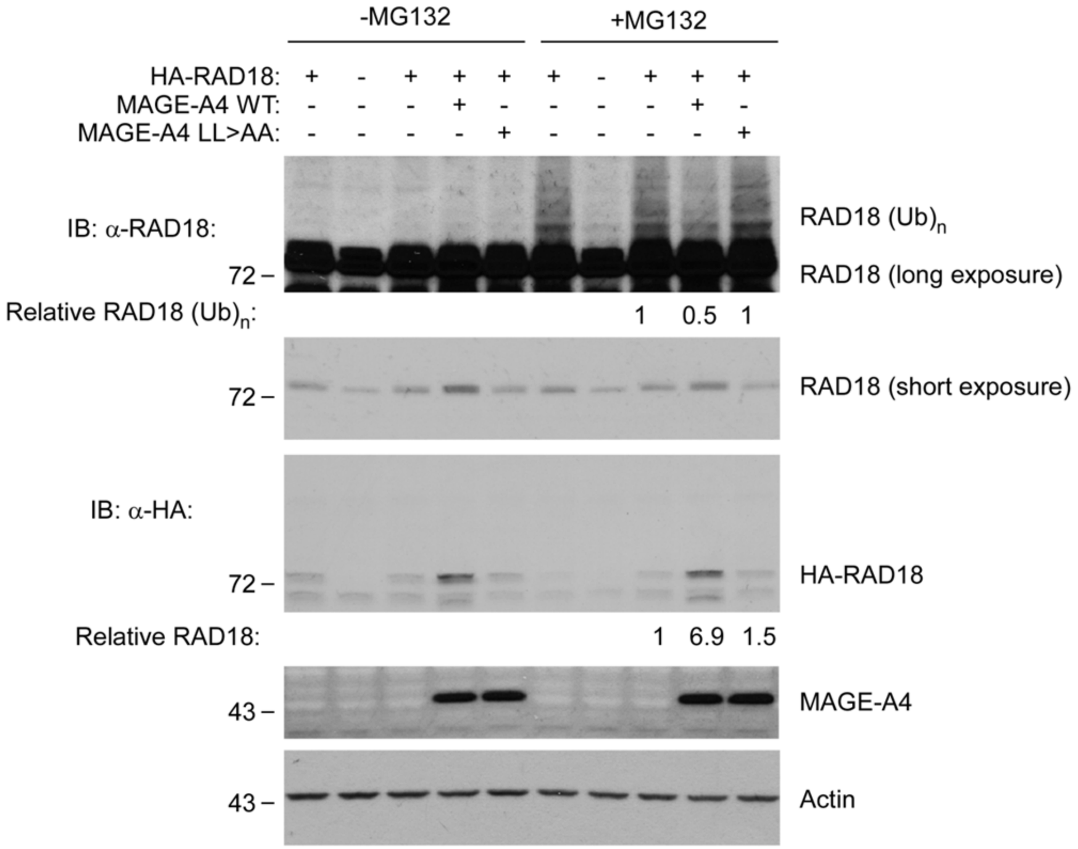


**Supplementary Fig. 4 Effect of MAGE-A4 on MG132-induced ubiquitin laddering of RAD18 in 293T cells.**

Replicate cultures of exponentially-growing 293T cells were co-transfected with expression vectors encoding HA-RAD18 and MAGE-A4 (WT or LL>AA mutant). 48 h later the resulting cultures were treated with MG132 (20 μM) for 4 h. Cell lysates were analyzed by immunoblotting with the indicated antibodies.

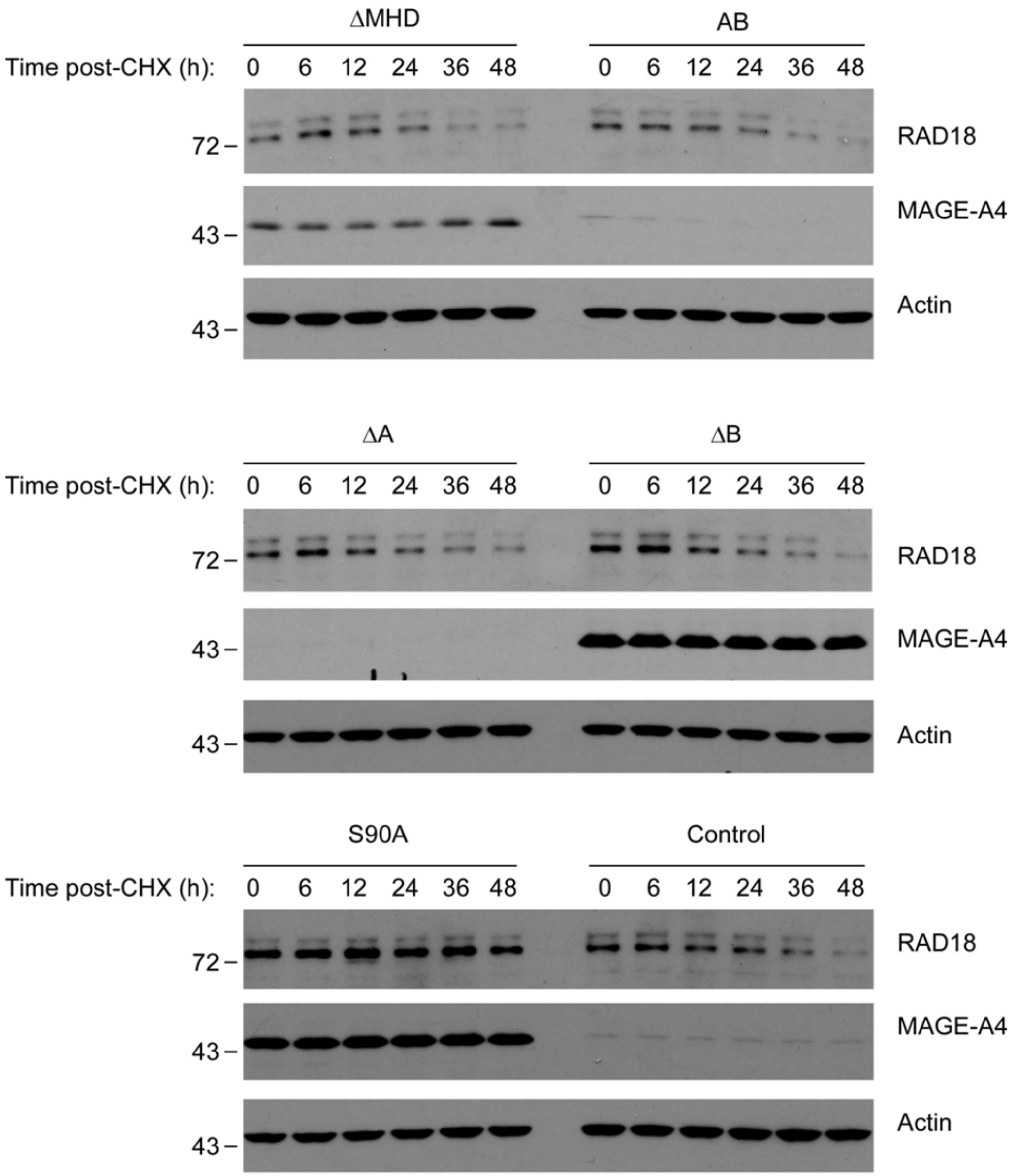


**Supplementary Fig. 5  Effect of different MAGE-A4 mutants on RAD18 stability.**

Replicate plates of 293T cells were transiently transfected with expression vectors encoding wild-type or mutant forms of MAGE-A4.  48 h post-transfection cells were treated with CHX and then harvested at different times post-CHX.  Cell extracts were analyzed by immunoblotting with antibodies against RAD18, MAGE-A4, and Actin.

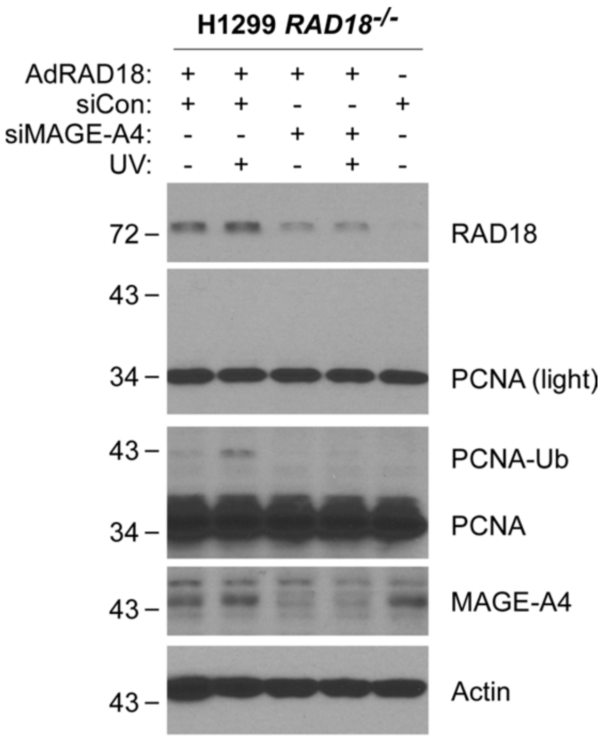


**Supplementary Fig. 6 MAGE-A4-depletion leads to reduced RAD18 expression and decreased PCNA mono-ubiquitination.**

*RAD18*$^{-/-}$ H1299 cells were transfected with MAGE-A4-directed siRNA or with a non-targeting siRNA control. 24 h post-transfection cells were complemented with adenovirally-encoded HA-RAD18. 24 h later, cells were UV-irradiated (20 J/m$^2$), or sham-treated for controls. 4 h after UV-treatment cells were lysed and extracts were analyzed by SDS-PAGE and immunoblotting with the indicated antibodies.

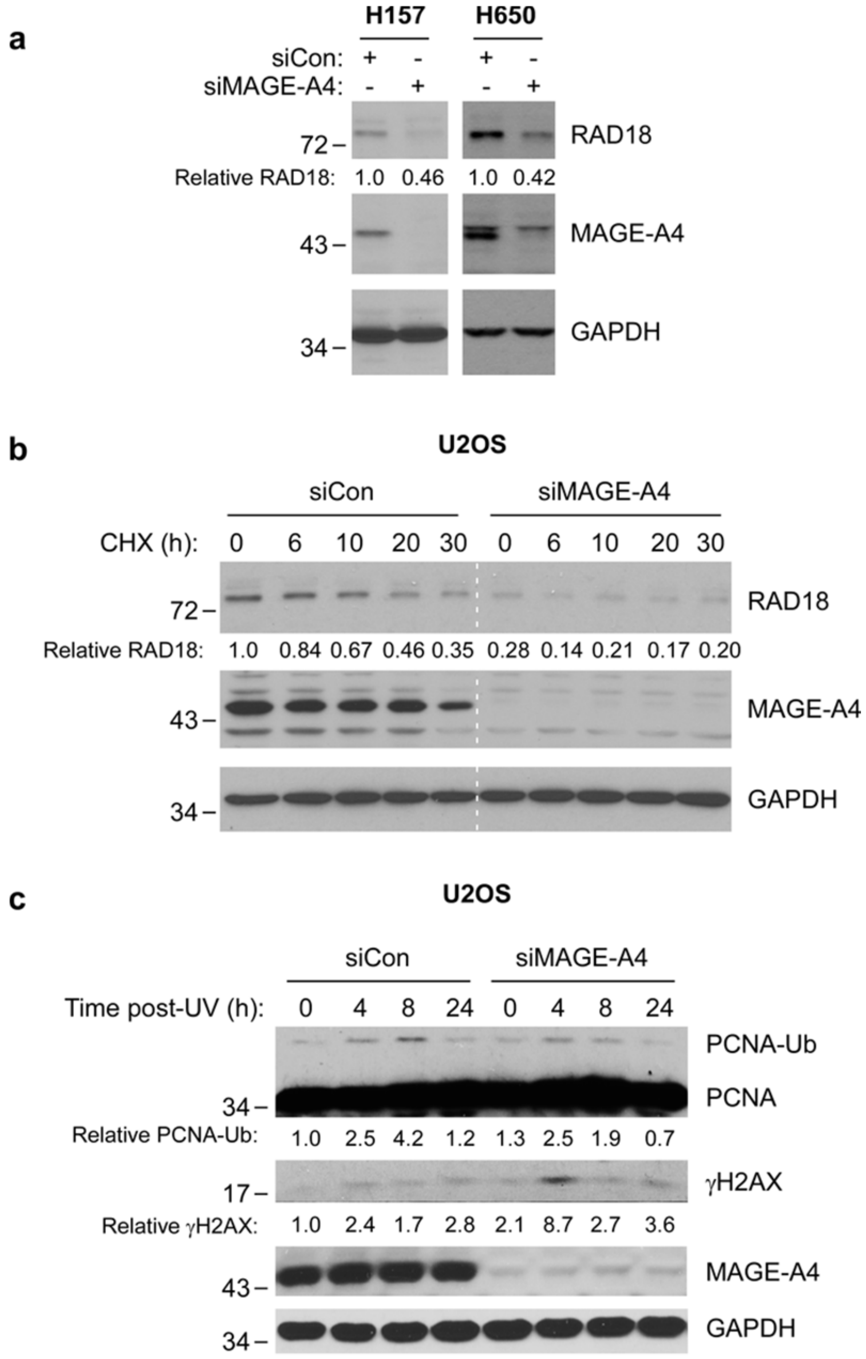


**Supplementary Fig. 7 Effect of MAGE-A4 depletion on RAD18 expression in H157, H650 and U2OS cells.**

(a) Exponentially-growing H157 and H650 cells were transfected with siMAGE-A4 or non-targeting control (siCon) siRNAs. 48 h later cells were lysed and extracts were analyzed by SDS-PAGE and immunoblotting with the indicated antibodies.

(b)-(c) Replicate plates of exponentially-growing U2OS cells were were transfected with siMAGE-A4 or non-targeting control (siCon) siRNAs. 48 h later, some cultures were treated with Cycloheximide (CHX, 100 μg/ml) to block new protein synthesis. At indicated times after CHX treatment, control and MAGE-A4-depleted cells were analyzed by SDS-PAGE and immunoblotting with antibodies against RAD18 and MAGE-A4 (b). Other replicate cultures were irradiated with UV-irradiated (5 J/$m^2$), or sham-treated for controls. At different times after UV-treatment, cells were harvested and analyzed by SDS-PAGE and immunoblotting using antibodies against PCNA and γH2AX.

a

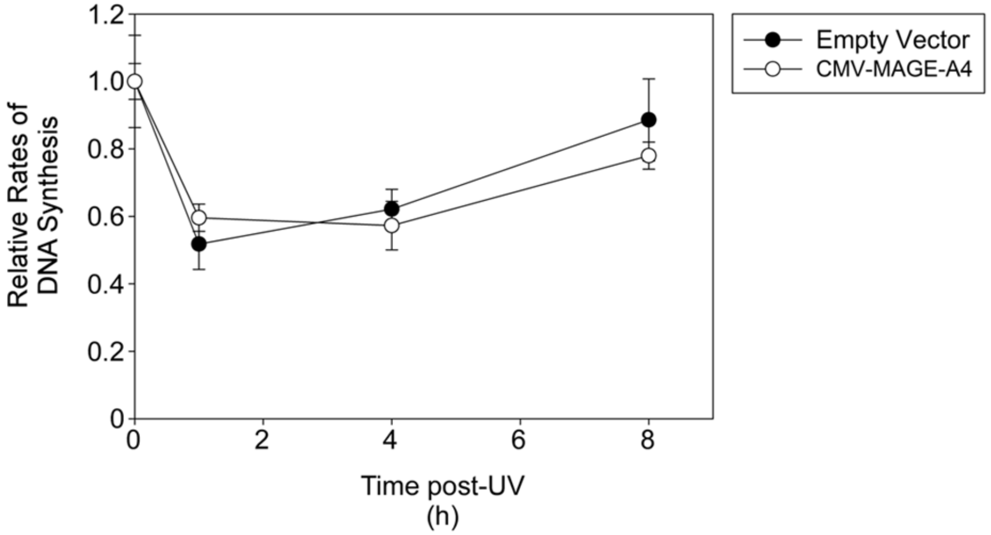


b

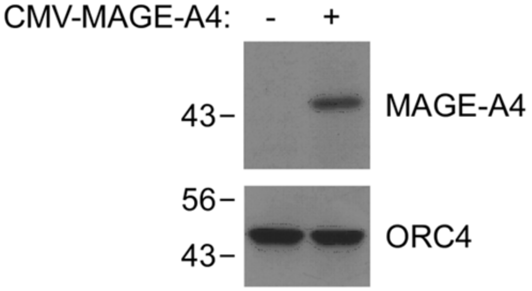


**Supplementary Fig. 8 MAGE-A4 expression in 293T cells does not affect inhibition or recovery of DNA synthesis after UV-irradiation.**

(a) Duplicate 10 cm plates of 293T cells were transfected with MAGE-A4 expression plasmid (CMV-MAGE-A4) or an empty vector for control. 24 h later the transfected cells were re-plated in 60 mm tissue culture dishes (for protein analysis) or 24-well plates (for DNA synthesis assays). The cells were UV-irradiated (5 $J/m^2$) or sham-treated and rates of DNA synthesis at various times post-UV were determined by measurements of [$^3$H]-thymidine incorporation. For each time point, rates of DNA synthesis were measured in triplicate and are normalized to DNA synthesis rates immediately prior to irradiation. Each data point represents the mean of the triplicate determinations and the error bars represent the range. (b) Coincident with UV-treatments, the 60 mm plates were used to prepare cell extracts for SDS-PAGE and immunoblotting to verify MAGE-A4 expression.

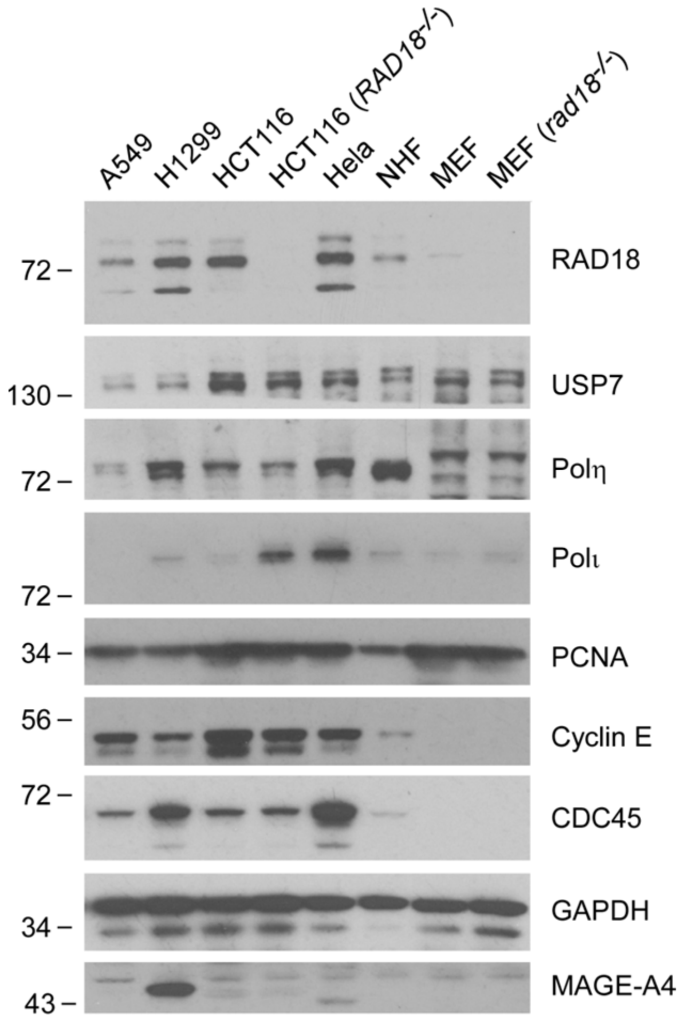


**Supplementary Fig. 9  Heterogeneous expression of MAGE-A4 and TLS proteins in commonly-studied cell lines.**

Extracts from different cell lines were subject to immunoblot analysis using antibodies against TLS proteins and cell cycle regulators as indicated.

**Supplementary Fig. 10 Uncropped Scans of Key Western Blots**

**Fig. 1e**

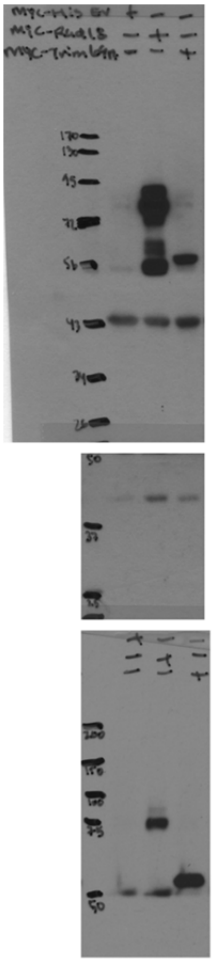

**Fig. 2c**

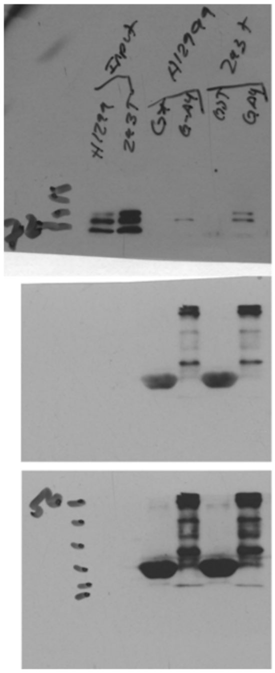


**Fig. 3b**

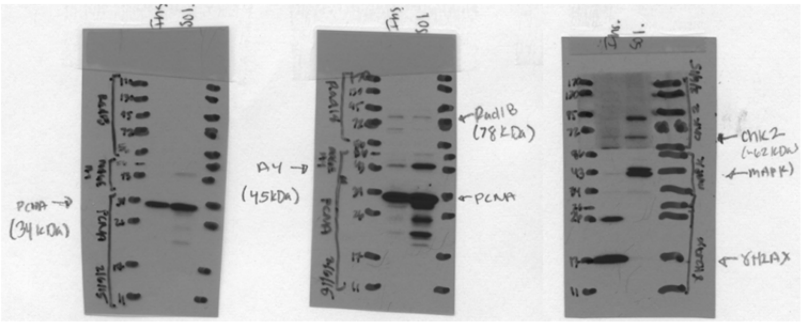

**Fig. 3d**

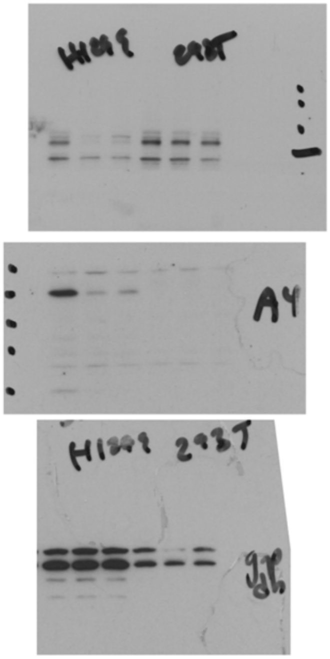


**Fig. 3e**

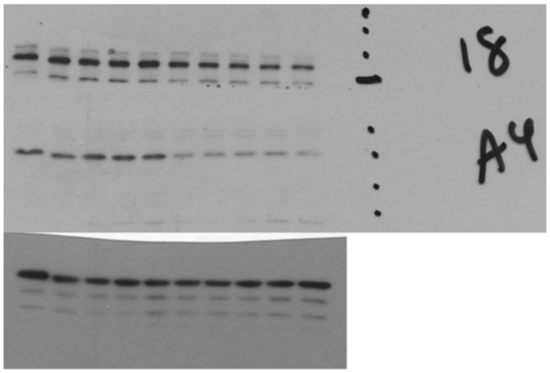


**Fig. 3i**

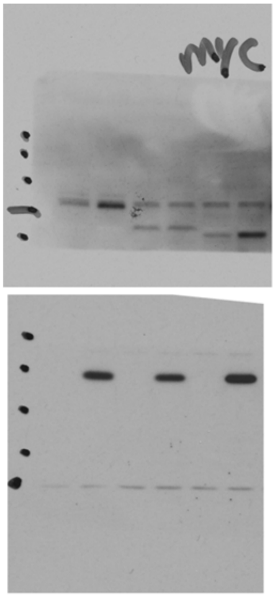


**Fig. 3j**

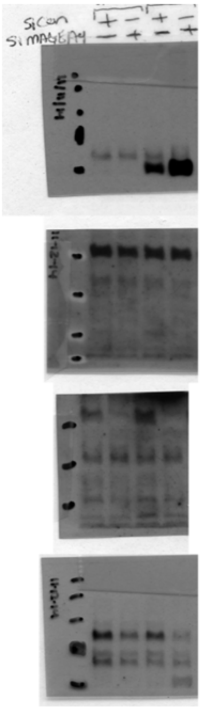

**Fig. 4**

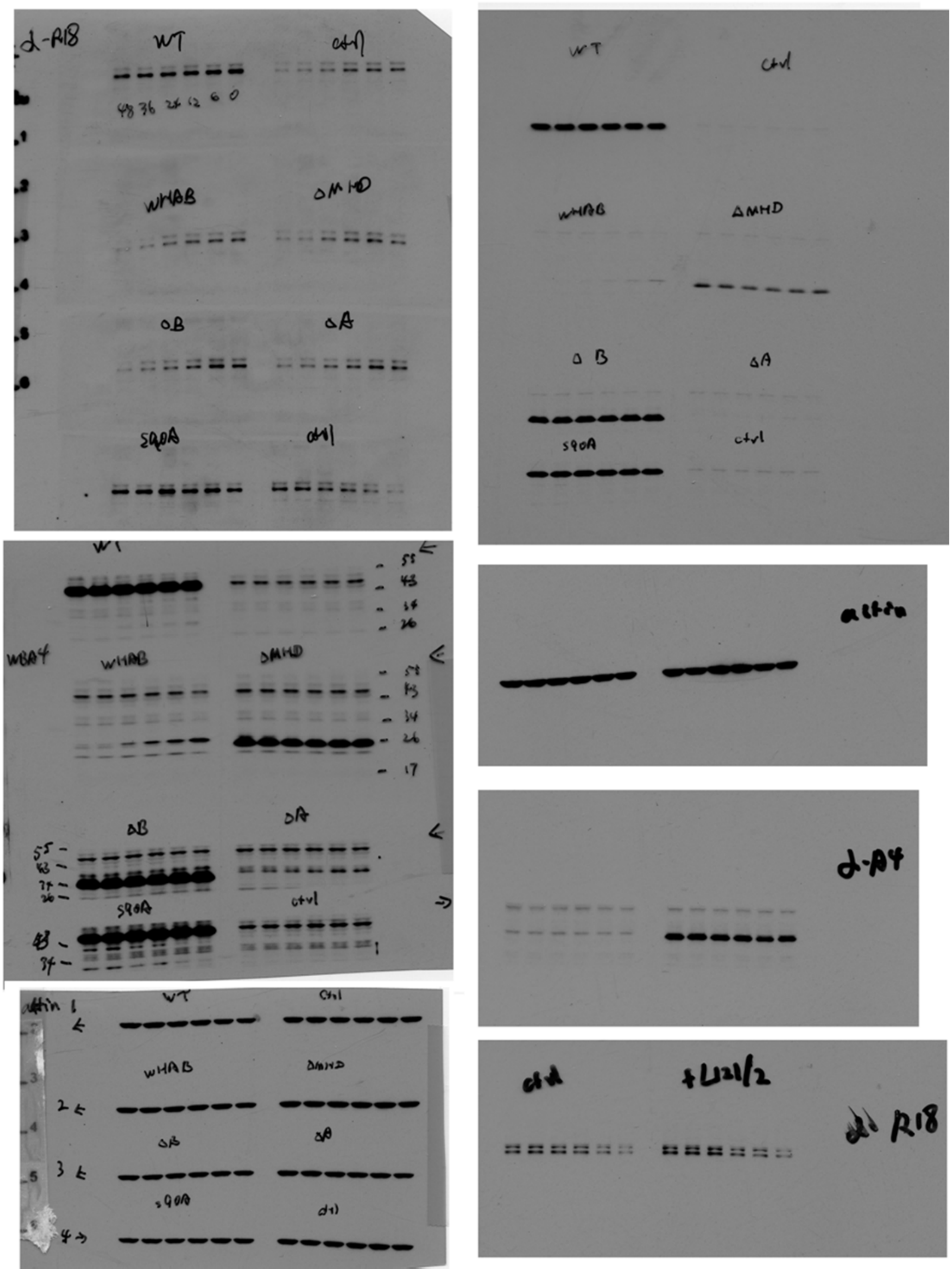

**Fig. 5**

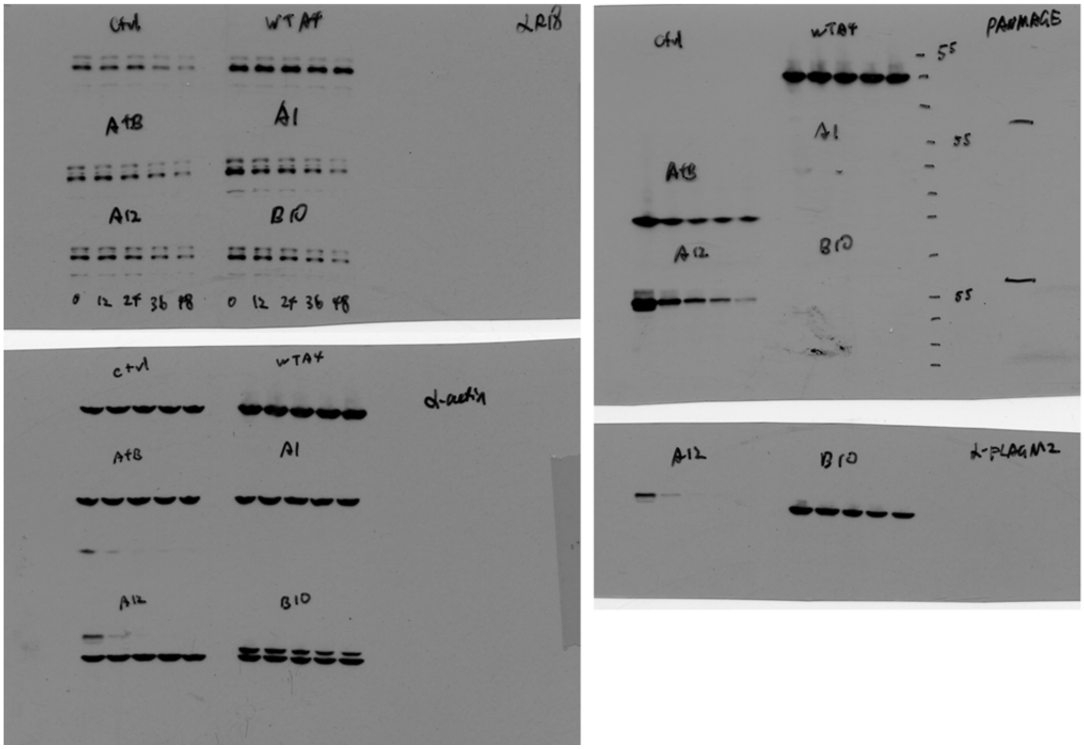


**Fig. 6a**

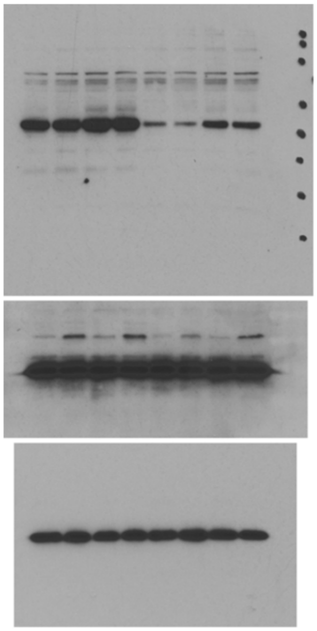

**Fig 6e**

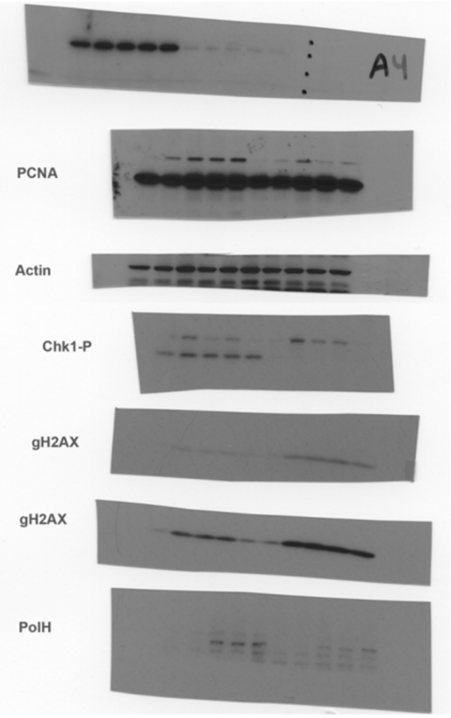


**Supplementary Fig. 4**

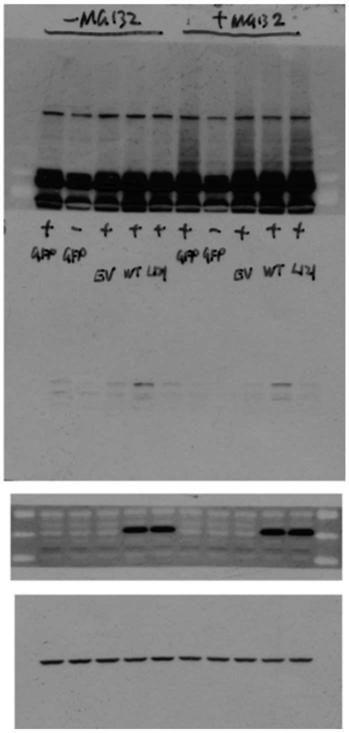


**Supplementary Fig. 6**

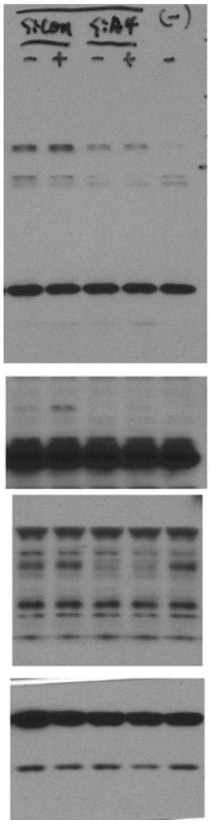

**Supplementary Note 1**

RAD18 Δ402-444 lacks a domain that promotes Polη-binding and facilitates association of the RAD18-Polη complex with PCNA. Unexpectedly however, Polη was detected in the immunopreciptated HA-RAD18 Δ402-444 complex while PCNA (but not Polη) was detected in the HA-RAD18 complex (see Supplementary Data 1).

The explanation for this apparent discrepancy lies in the low sensitivity of the mass spectrometry. Mutation of the Polη-binding domain of RAD18 leads to a ~50% decrease in Polη- RAD18 complex formation.[1] Mass spectrometry has a limited dynamic range and is not sufficiently quantitative for measuring 2-fold changes - particularly for a low abundance protein like Polη which is ~75x less abundant than RAD18.[2]

Moreover, since Polη is low-abundance protein, it fell outside of the dynamic range for the immunoprecipitated WT RAD18 (maxquant intensity supports this). Based on intensity, ~ 2x more RAD18 was detected in the WT HA-RAD18 samples as compared to the RAD18 Δ402-444 mutant (again, based on maxquant intensities). Because HA-RAD18-WT was expressed at higher levels than the RAD18 Δ402-444 mutant, and because the Polη-RAD18 interaction was already saturated (due to limiting levels of Polη), the amount of Polη relative to RAD18 was apparently less in WT RAD18 immunoprecipitates when compared to the RAD18 Δ402-444 mutant. That is, due to the greater dynamic range in the WT RAD18 sample, less Polη was detected. In the context of this report, the mass spectrometry experiment serves solely to document our initial observation that RAD18 associates with MAGE-A4 - an interaction that is independently validated in many other experiments in the 'Results'.

**Supplementary References**

1. Day, T. A. et al. Phosphorylated Rad18 directs DNA polymerase eta to sites of stalled replication. *J Cell Biol* **191,** 953-966, doi:10.1083/jcb.201006043 (2010).

2. Durando, M., Tateishi, S. & Vaziri, C. A non-catalytic role of DNA polymerase eta in recruiting Rad18 and promoting PCNA monoubiquitination at stalled replication forks. *Nucleic Acids Res*, doi:10.1093/nar/gkt016 (2013).
